# Natural variation at *XND1* impacts root hydraulics and trade-off for stress responses in *Arabidopsis*

**DOI:** 10.1038/s41467-018-06430-8

**Published:** 2018-09-24

**Authors:** Ning Tang, Zaigham Shahzad, Fabien Lonjon, Olivier Loudet, Fabienne Vailleau, Christophe Maurel

**Affiliations:** 1 BPMP, CNRS, INRA, Montpellier SupAgro, Université de Montpellier, 34060 Montpellier, France; 2 LIPM, Université de Toulouse, INRA, CNRS, 31326 Castanet-Tolosan, France; 3 Institut Jean-Pierre Bourgin, INRA, AgroParisTech, CNRS, Université Paris-Saclay, 78000 Versailles, France; 4 Present Address: Institute of Molecular, Cell and Systems Biology, College of Medical, Veterinary and Life Sciences, University of Glasgow, Bower Building, Glasgow, G12 8QQ UK

## Abstract

Soil water uptake by roots is a key component of plant performance and adaptation to adverse environments. Here, we use a genome-wide association analysis to identify the XYLEM NAC DOMAIN 1 (XND1) transcription factor as a negative regulator of *Arabidopsis* root hydraulic conductivity (*L*p_r_). The distinct functionalities of a series of natural *XND1* variants and a single nucleotide polymorphism that determines XND1 translation efficiency demonstrate the significance of *XND1* natural variation at species-wide level. Phenotyping of *xnd1* mutants and natural *XND1* variants show that XND1 modulates *L*p_r_ through action on xylem formation and potential indirect effects on aquaporin function and that it diminishes drought stress tolerance. XND1 also mediates the inhibition of xylem formation by the bacterial elicitor flagellin and counteracts plant infection by the root pathogen *Ralstonia solanacearum*. Thus, genetic variation at *XND1*, and xylem differentiation contribute to resolving the major trade-off between abiotic and biotic stress resistance in *Arabidopsis*.

## Abstract (web summary)

Soil water uptake is a major determinant of plant performance and stress tolerance. Here the authors show that, by affecting xylem formation in the root, natural variation at the *Arabidopsis XND1* locus has contrasting effects on root hydraulics and drought tolerance versus pathogen resistance.

## Introduction

The growth performance and survival of terrestrial plants, whether in favorable or adverse environments, crucially depend on a proper uptake and management of water. Most plant species forage the soil for water through continuous growth and development of roots into a ramified architecture. The intrinsic water transport properties of root tissues (i.e., their root hydraulic conductivity, *L*p_r_) are also important for efficient uptake and transfer of water towards the shoots. *L*p_r_ shows a high environmental plasticity, with typical regulations depending on the availability of water, mineral nutrients or oxygen in the soil^1^. The same stimuli act on root growth and development, thereby altering root system architecture (RSA)^2^. Overall, growth and water transport properties of roots, which combine into the so-called root hydraulic architecture, determine the plant’s capacity to capture soil water under changing or heterogeneous soil conditions. Plants also display remarkable intraspecific genetic variations in RSA and hydraulics^3–7^, with possible impacts on abiotic stress responses. Thus, the ultimate question is to understand how combined genetic and physiological adjustments of RSA and root water permeability contribute to plant adaptation to specific habitats or climatic scenarios.

Root water transport per se relies on several fundamental processes, often presented as sequential. Radial water flow, from the soil to the vasculature in the stele, is mediated through cell walls (apoplastic path) or from cell-to-cell. The latter path combines transcellular (across cell membranes and aquaporins) and symplastic (across plasmodesmata) transport. Water is then axially transported to the aerial parts through xylem vessels. The contexts in which root xylem can be hydraulically limiting are still debated^8^. Based on Poiseuille’s law of laminar flow, it was calculated that under water sufficient conditions this tissue is supposedly not limiting with respect to root structures mediating radial transport^9^. This may not be true in root tips, whereby vessels are not fully differentiated. Xylem cavitation under drought can also dramatically reduce plant hydraulic conductance and confer high plant vulnerability^10^. Conversely, drought impacts xylem differentiation further supporting a crucial role of vascular transport in these conditions^5,8^. However, this view may not apply to certain species or under mild water stress since intraspecific variation of xylem size in major crops such as rice was not associated to any growth advantage, especially under water deficit^11^. Thus, the links that xylem vessel differentiation establishes between root growth and development, hydraulics, and stress responses are not fully established^8^. In contrast, detailed studies have revealed how positioning of xylem axes contributes to early vascular pattern formation and how subsequent differentiation of xylem tracheary elements occurs through cell clearance by programmed cell death and deposition of lignin in secondary cell walls^12^. These developmental processes are controlled by regulatory networks involving NAC (NAM, ATAF1,2, and CUC2)^13^ and MYB (myeloblastosis)-type transcription factors^14^. The former proteins have played a key evolutionary role in water-conducting structures from moss to vascular plants^15^.

While root hydraulics is prone to fine biophysical analyses, genetic dissection of this trait has been somewhat lagging due in part to technical difficulties in defining proper hydraulic phenotypes. Reverse genetic analyses of candidate genes have uncovered the limiting role of aquaporins^1^ or endodermal barriers^16,17^. In contrast, other components which determine root anatomy or environmental signaling and which can potentially interfere with root hydraulic properties have been poorly explored^18^. Quantitative genetics approaches based on intraspecific variations of root hydraulics^3,4^ could help uncover such molecular components. In line with these ideas, quantitative trait locus (QTL) analysis of *L*p_r_ in a biparental recombinant population (Bur-0 × Col-0) of *Arabidopsis* led to the cloning of *hydraulic conductivity of root 1*, a Raf-like MAPKKK gene that acts as a negative regulator of *L*p_r_^19^. Here, we perform a genome-wide association analysis as another approach to identify genes controlling root hydraulics in *Arabidopsis*. We identify *XYLEM NAC DOMAIN 1* (*XND1*) as a key negative regulator of *Arabidopsis* root hydraulics at the species-wide level. Our study also reveals how genetic variation at *XND1* may contribute to the trade-off between abiotic stress tolerance and biotic defense in *Arabidopsis*.

## Results

### A GWA study uncovers two novel genes controlling *L*p_r_

**Fig. 1:**
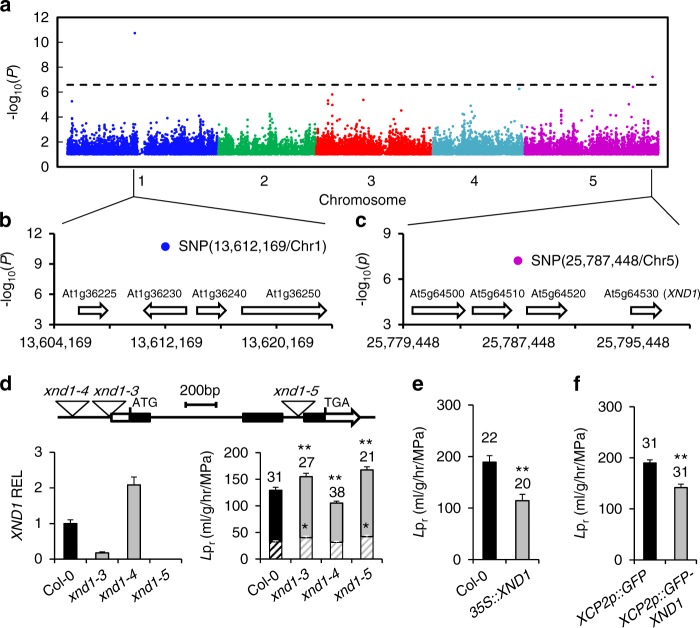
Identification of *XND1* as a genetic determinant of root hydraulic conductivity. **a** Manhattan plot of GWA for root hydraulic conductivity (*L*p_r_) data based on a conditioned accelerated mixed-model. The chromosomes are depicted in different colors and the *x*-axis represents the chromosome number and position. A Bonferroni corrected significance threshold at *α* = 0.05 is indicated by the horizontal dashed line. **b**, **c** Gene models of 20-kb genomic regions surrounding the two most significantly associated SNPs. The blue and purple dots represent these SNPs, and numbers on *x-*axes indicate chromosomal positions in base pairs. **d** Molecular and phenotypic characterization of three *xnd1* T-DNA insertion lines. The upper diagram shows a schematic representation of the *XND1* genomic region with positions of the three T-DNA insertions. The bar graph on the left shows *XND1* transcript abundance relative to Col-0 (relative expression level, REL) in the *xnd1* lines (means ± SE, based on two biological repeats). The bar graph on the right shows *L*p_r_ phenotypes before (whole bars) or after (hatched bars) root treatment for 30 min with the aquaporin blocker NaN_3_. **e**, **f** *L*p_r_ phenotype of Col-0 transgenic lines with *XND1* constructs under control of a *CaMV35S* (**e**) or a xylem specific *XCP2Pro* (*XCP2p*) (**f**) promoter. In **f**, plants expressing GFP alone under the control of *XCP2Pro* were used as negative controls. In all panels, *L*p_r_ data (means ± SE) were based on the indicated number of plants in two to three independent experiments. Asterisks indicate significant differences with respect to control lines (Student’s *t* test; ^*^P < 0.05, ^**^P < 0.01)

A set of 143 *Arabidopsis* accessions from the RegMap panel^20^ was phenotyped for root hydraulics (Supplementary Data 1). A fourfold variation of *L*p_r_ was observed among accessions (Supplementary Figure 1), with a coefficient of variation of 0.245 and a broad-sense heritability *h*^2^ = 0.36. Conditional genome wide association (GWA) mapping using 250k single nucleotide polymorphisms (SNP) data^20^, and an accelerated mixed-model algorithm method with four markers as cofactors^21^ revealed two SNPs that were significantly associated with *L*p_r_ variation (Bonferroni multiple testing correction at *α* = 0.05) and contributed to 18.3% and 7.3% of the genetic variance, respectively (Fig. 1a). One was located on chromosome (Chr) 1 (position 13,612,169), while the other was on Chr 5 (position 25,787,448). Considering 20-kb genomic regions surrounding these two association SNPs (Fig. 1b, c), we identified eight candidate genes for *L*p_r_ variation. None of these genes showed SNPs that were in strong linkage disequilibrium (LD) with the corresponding GWA studies (GWAS) peak SNP (Supplementary Figure 2). They were therefore further evaluated using a total of 15 Col-0-derived T-DNA insertion lines. As for the Chr1 region, two allelic insertion lines for At1g36240 showed, with respect to Col-0, an increase in *L*p_r_ by 11 or 17%, while mutant lines for three neighboring genes did not show any significant *L*p_r_ phenotype (Supplementary Table 1). When considering the four genes located in the Chr 5 region, we observed significantly altered *L*p_r_ specifically for three T-DNA insertion lines of At5g64530 (Supplementary Table 1; Fig. 1d). The data reveal the power of GWA mapping for identifying genetic determinants of specific traits like root hydraulics. At1g36240 encodes a putative ribosomal protein which will be investigated in another work. At5g64530 encodes Xylem NAC Domain 1 (XND1), a NAC transcription factors that antagonizes xylem differentiation, by negatively regulating secondary cell wall synthesis and programmed cell death^22^. Its putative function in axial water transport led us to examine in closer details its contribution to root hydraulics, which has not been described previously.

### XND1 negatively regulates root hydraulics

Quantitative reverse transcription polymerase chain reaction (qRT-PCR) analyses revealed contrasting *XND1* mRNA abundance in the three *xnd1* allelic mutants (Fig. 1d). *xnd1–3* and *xnd1–4*, which both exhibit a T-DNA insertion in the *XND1* promoter region appeared as knock-down and activation lines, respectively. Consistent with a T-DNA insertion in the second intron, *xnd1–5* can rather be considered as a knock-out allele. When considering the root dry weight (DW), primary and total root length, or lateral root density in plants grown in hydroponics, all three *xnd1* genotypes had a root architecture similar to that of Col-0 (Supplementary Figure 3). In contrast, and by reference to Col-0, *L*p_r_ was increased in hypofunctional mutants (*xnd1–3*, *xnd1–5*) by up to 29.7 ± 7.6% and decreased by 18.6 ± 4.7% in the *xnd1–4* overexpression mutant. These results indicate that XND1 acts as a true negative regulator of root hydraulics. Consistent with this, overexpression of *XND1* under the control of a *CaMV35S* promoter (Supplementary Figure 4A, B) resulted in a dramatic (−39.5 ± 7.7%) reduction in *L*p_r_ (Fig. 1e). XND1 is expressed in root xylem, preferentially in association with differentiating tracheary elements^22,23^. Transgenic expression of a GFP–XND1 fusion protein under the control of xylem specific promoter *XCP2Pro* (Supplementary Figure 4C, D) reduced *L*p_r_ by 25.3 ± 4.3% (Fig. 1f), implying that XND1 acts in vascular tissues to exert hydraulic effects.

### Allelic diversity at *XND1* validates GWA analyses

**Fig. 2:**
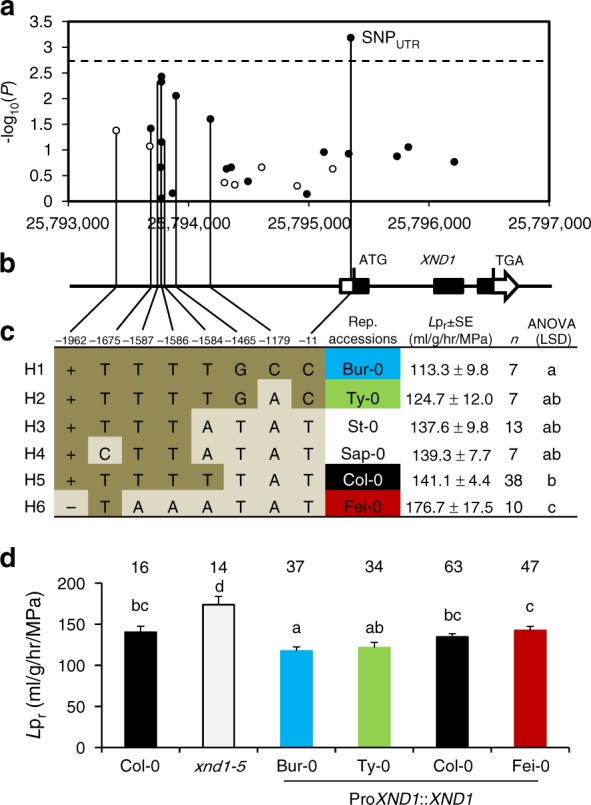
A *XND1*-based association analysis allows refining the natural allelic variations of *XND1*. **a** The association with *L*p_r_ of 27 polymorphic sites (MAF > 0.05) in the indicated *XND1* genomic region was investigated in a set of 112 accessions. The *x*-axis shows the nucleotide position of each variant, with empty and filled circles indicating INDELs and SNPs, respectively. The *y*-axis shows the –log_10_(*P*) for the association tests, with the significance threshold at *α* = 0.05 indicated with a dashed line. **b** The eight polymorphisms selected for further analysis are projected onto a schematic representation of *XND1* gene structure. For position −1962, + and − represent an insertion and deletion, respectively. The boxes represent exons, with solid and empty boxes showing translated and untranslated regions, respectively. The SNP at Chr 5-P25,795,349 that surpassed the significance threshold in **a** is located in the 5’-UTR of *XND1* and indicated as SNP_UTR_. **c** The eight selected nucleotide polymorphisms (with their distance from translation start site shown on the top) define six haplogroups (H1–H6). Representative accessions and mean *L*p_r_ ± SE within each haplogroup (*n*, accessions number) are shown. One-way ANOVA (Fisher’s LSD, *P* < 0.05) was used to test the significance of the *L*p_r_ data. **d** Transgenic complementation of *xnd1–5* with different allelic forms of *XND1*. The *L*p_r_ of Col-0, *xnd1–5*, and *xnd1–5* plants expressing *XND1* genomic fragments from Bur-0, Ty-0, Col-0, or Fei-0, was tested using 3–5 independent transgenic lines per *XND1* allele (see Supplementary Figure 5). Mean values ± SE (*n* = 14–63 plants) are shown with sample size indicated on the top. One-way ANOVA (Fisher’s LSD, *P* < 0.05) was used to test the significance of the data

The *L*p_r_ associated SNP identified at position 25,787,448/Chr 5 during GWA analysis is located 7.9 kb apart from the *XND1* coding region, whereas SNPs that are closer did not show any significant association. This hints to possible allelic heterogeneity and multiple mutations in *XND1* that would contribute to *L*p_r_ variation and are linked to the associated SNP. Newly released genomic sequences^24^ were used to evaluate in closer details natural variation at *XND1* among 112 accessions, of which 85 belong to the initially investigated panel while the remaining corresponds to accessions for which phenotypic data were generated later (Supplementary Data 2). Considering a genomic region from 2 kb upstream to 0.3 kb downstream of the *XND1* coding region, we used a generalized linear model to test the association with *L*p_r_ at 27 polymorphic sites (minor allele frequency (MAF) > 0.05), including SNPs and INDELs (Fig. 2a, b). One single SNP located at position 25,795,349 in the 5′-UTR of *XND1* surpassed the significance threshold and was thereafter named SNP_UTR_. Several SNPs also pointed to possible associations in the promoter region, consistent with putative allelic heterogeneity. Further, we used the eight polymorphisms showing the lowest *P* values (*P* < 0.075) in this association analysis to define six haplogroups, each containing 7–38 accessions (Fig. 2c; Supplementary Data 2). These haplogroups fall into four phenotypic classes with significant differences in mean *L*p_r_ values (ANOVA; *P* < 0.05) (Fig. 2c) suggesting that genetic diversity at *XND1* truly contributes to shaping *L*p_r_ variation at the species level. To establish this point further, we selected four accessions (Bur-0, Ty-0, Col-0, and Fei-0) as representative members of haplogroups with contrasting mean *L*p_r_ values (Fig. 2c). Corresponding genomic fragments encompassing *XND1* were introduced into *xnd1–5* and tested for their capacity to complement the mutant *L*p_r_ phenotype (Fig. 2d). In these experiments, we used a set of three to five independent transgenic lines per haplotype, which provide mean *XND1* expression levels similar to that in native Col-0 (Supplementary Figure 5A,B). All four complemented series exhibited lower *L*p_r_ than *xnd1–5*, indicating that each of *XND1* allelic forms was functional in a Col-0 background (Fig. 2d; Supplementary Figure 5C; Supplementary Data 3). In addition, transformation with the Bur-0 or Ty-0 alleles yielded lower *L*p_r_ values than when using the Col-0 or Fei-0 allele, or the Fei-0 allele, respectively. Thus, with respect to Col-0 and Fei-0, the first two accessions harbor somewhat hyperfunctional alleles.

To further investigate the significance of variation at *XND1*, we revisited previous phenotyping data from a Bur-0 × Col-0 cross^19^. Although there was no sign of a purely additive QTL at the bottom of Chr 5 in this cross^19^, we detected an epistatic QTL controlling *L*p_r_ variation (Supplementary Figure 6), possibly including *XND1* effect. The phenotypic consequence of segregation at *XND1* region is conditioned by the allele present at a locus on Chr 2, suggesting that phenotypic expression of *XND1* variation may depend on the genetic background. We note that, consistent with the effects of introducing a Bur-0 *XND1* allele in Col-0 (Fig. 2d), the RILs fixed for Bur-0 at *XND1* region and Col-0 at the Chr2 locus exhibited a reduced *L*p_r_ (Supplementary Figure 6).

The overall genetic data validate the identification of *XND1* by GWA mapping, and support the idea that allelic differences at *XND1* contribute to the natural variation of *L*p_r_ in *Arabidopsis*.

### A SNP in *XND1* can account for natural variation of *L*p_r_

**Fig. 3:**
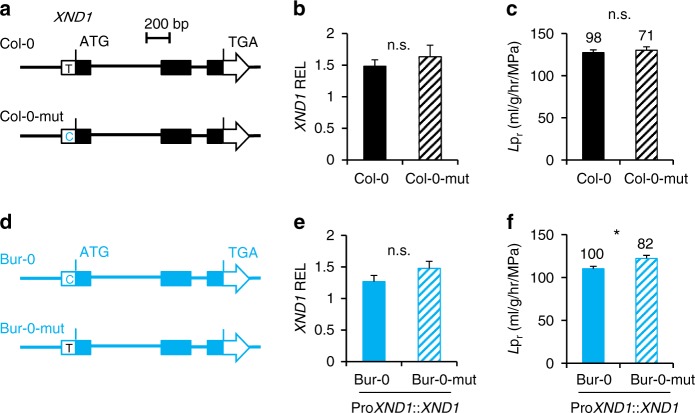
SNP_UTR_ of *XND1* contributes to *L*p_r_ variation. Transgenic complementation of *xnd1–5* with Col-0 or Bur-0 allelic forms of *XND1*, either wild-type or with a site-directed mutation at SNP_UTR_. **a**, **d** Schematic representation of investigated *XND1* allelic forms. Col-0 and Bur-0 indicate the wild-type forms while Col-0-mut and Bur-0-mut indicate the corresponding point mutated forms. **b**, **e** Transcript abundance of *XND1* in *xnd1–5* homozygous transgenic lines expressing the Col-0, Col-0-mut, Bur-0, or Bur-0-mut forms of *XND1*. Three to five independent transgenic lines (two biological replicates for each) were studied for each form. Mean values ± SE (*n* = 18–30) were normalized to transcript abundance in Col-0. **c**, **f** *L*p_r_ of the transgenic lines described above. Three to five independent transgenic lines were phenotyped for each form. Mean values ± SE are shown with total number of plants indicated on the top. Student’s *t* test (^*^P < 0.05) was used to assess the statistical significance of the data (n.s. = not significant). *XND1* expression and *L*p_r_ data in individual transgenic lines are shown in Supplementary Figure 7

Among the 112 accessions analyzed above, the SNP_UTR_, with C vs. T variation, identifies two haplogroups with significantly different *L*p_r_ (SNP_UTR_-C, *L*p_r_ = 114.8 ± 7.6, *n* = 16; SNP_UTR_-T, *L*p_r_ = 145.5 ± 3.3, *n* = 96; *P* < 0.01). Bur-0 and Col-0 belong to the first and second haplogroups, respectively (Fig. 2c). We addressed the functional significance of SNP_UTR_ by transgenic complementation of *xnd1–5* mutant with Bur-0 or Col-0 allelic forms of *XND1*, either native or with a converting point mutation at SNP_UTR_ (Fig. 3a, d). While mutation of the Col-0 allele did not influence *L*p_r_ (Fig. 3b, c; Supplementary Figure 7A, C; Supplementary Data 4), the conversely mutated Bur-0 allele conferred a higher *L*p_r_ than its native counterpart in *xnd1–5* complemented lines (Fig. 3e, f; Supplementary Figure 7B, D; Supplementary Data 4). These data establish that SNP_UTR_ contributes to natural variation of XND1 function. Since *L*p_r_ phenotypic differences induced by these point mutations were in either case smaller than when comparing the two wild-type alleles (Fig. 2d; Fig. 3c, f), we hypothesize that other polymorphisms act, independently or in interaction with SNP_UTR_, to induce the full allelic effects. Effects of these polymorphisms would be more pronounced in the Col-0 allele than in the Bur-0 allele.

**Fig. 4:**
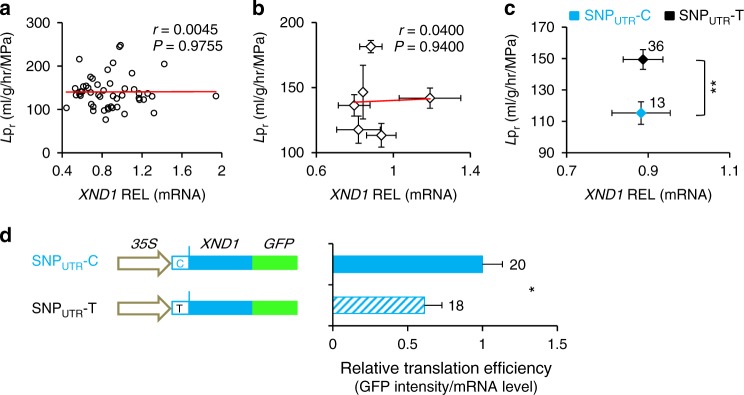
*XND1*-dependent natural variation of *L*p_r_ is contributed by effects of SNP_UTR_ on XND1 translation. **a**–**c** *XND1* transcript abundance was measured in the roots of 49 accessions using 6–10 plants and two independent experiments per accession. Data were analyzed considering each individual accession (**a**) or their grouping in the H1-H6 (**b**) or SNP_UTR_-C vs. SNP_UTR_-T haplogroups (**c**). Pearson correlation coefficient (*r*) and *P* value (*P*) between *XND1* transcript level and *L*p_r_ are shown in **a** and **b**. Error bars in **b** and **c** indicate SE. Accession number per haplogroup was *n* = 3–18 in **b** and as indicated in **c** (^**^P < 0.01 in Student’s *t* test). **d** A XND1–GFP fusion construct was placed downstream of a *CaMV35S* promoter and a *XND1* 5’-UTR, with either C or T at SNP_UTR_. Note that this SNP is the only variation between Bur-0 and Col-0 *XND1* alleles present in the construct sequence. Transcript abundance and fluorescence intensity of XND1–GFP was quantified in the roots of 9–10 transgenic lines per genotype, with two biological replicates. Relative translation efficiency of XND1–GFP was calculated in each individual line from the ratio of fluorescence to mRNA abundance. Mean values ± SE based on total number of lines and repeats are indicated on the right and were normalized to the data for SNP_UTR_-C. Student’s *t* test (^*^P < 0.05) was used to assess the statistical significance of the data

Next, we addressed the general molecular mechanisms that can account for natural variation of XND1 function. Knowing that the *XND1* allelic variation pointed by GWA and validated by transgenic complementation does not reside in the *XND1* coding sequence, we investigated natural variation in *XND1* expression and its potential functional significance. The abundance of *XND1* transcripts was measured in the roots of 49 randomly selected accessions representative of the six previously defined H1–H6 haplogroups. No correlation of *L*p_r_ with *XND1* transcript abundance was observed when considering all individual accessions (Fig. 4a), or their grouping in the H1–H6 (Fig. 4b) or SNP_UTR_-C vs. SNP_UTR_-T (Fig. 4c) haplogroups. Thus, the SNP_UTR_ and other putative SNP(s) causing *L*p_r_ variations have marginal effects, if any, on mRNA abundance at this scale. To investigate putative effects of SNP_UTR_ on XND1 protein translation, a XND1-GFP fusion was placed under the control of a *CaMV35S* promoter and a *XND1* 5′-UTR, in its SNP_UTR_-C or SNP_UTR_-T form, and expressed in transgenic *xnd1–5* (Supplementary Figure 8). Based on 9–10 lines per genotype with overall similar transcript abundance, we used GFP fluorescence as a reporter of XND1–GFP accumulation (Supplementary Figure 9A–D). For each line, a relative translation efficiency was deduced from the ratio of XND1–GFP protein and mRNA abundances (Supplementary Figure 9E). Combined data (Fig. 4d) revealed that relative translation efficiency of the SNP_UTR_-C form was 63% higher than that of its SNP_UTR_-T counterpart. The positive effect of SNP_UTR_-C on XND1 translation and accumulation is consistent with lower *L*p_r_ in the corresponding haplogroup.

### XND1 affects both xylem formation and aquaporin activity

**Fig. 5:**
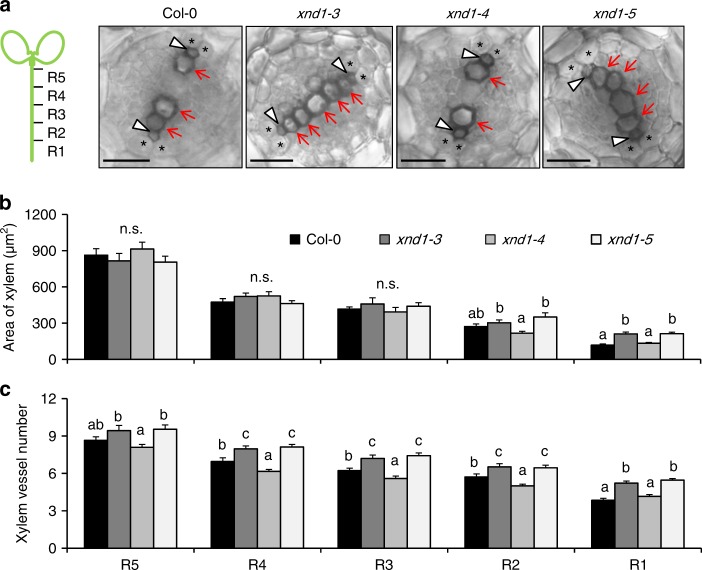
XND1 negatively regulates root xylem formation. **a** The figure shows schematic representation of 2-cm-long segments (R1–R5) used for characterization of xylem anatomy in the indicated genotypes and representative cross-sections within the R2 segment. Arrowheads and arrows indicate protoxylem and metaxylem, respectively. Asterisks indicate pericycle cells adjacent to xylem poles. Scale bars represent 20 µm. **b**, **c** Xylem area (**b**) and vessel number (**c**) (mean values ± SE) were measured in the indicated root segment and genotype, in 20–61 sections from 10–15 plants and 2–3 independent experiments. One-way ANOVA (Fisher’s LSD, *P* < 0.05) was used to test the significance of the data (n.s. = not significant)

To understand further the causes of *XND1*-mediated variation of *L*p_r_, we used sodium azide (NaN_3_), a common blocker of plant plasma membrane aquaporins^3,19^. NaN_3_ dramatically reduced *L*p_r_ in Col-0 and three T-DNA insertion *xnd1* genotypes, yielding a residual *L*p_r_, which was higher in loss-of-function alleles (*xnd1–3*, *xnd1–5*) than in wild type (Col-0) or gain-of-function (*xnd1–4*) plants (Fig. 1d). NaN_3_-resistant *L*p_r_ accounts for aquaporin-independent water transport pathways, whether radial (apoplasm) or axial (xylem vessels). Here, facilitation of NaN_3_-resistant pathways in *xnd1–3* and *xnd1–5* is consistent with XND1 acting as a negative regulator of xylem differentiation. To assess this point, we probed xylem morphology along the root axis by sampling tissues in 2-cm-long consecutive segments from the root tip (Fig. 5). By comparison to Col-0, formation of the xylem (especially metaxylem) was advanced in *xnd1–3* and *xnd1–5*, while delayed in *xnd1–4* (Fig. 5a). Specifically, overall xylem area was enhanced in *xnd1–3* and *xnd1–5* in the most apical segment (R1) (Fig. 5b). Xylem vessel number was consistently increased in several segments (R1–R4) of loss-of-function *xnd1* alleles whereas it was reduced in the R2–R4 segments of *xnd1–4* (Fig. 5c).

We also observed that the absolute difference in *L*p_r_ between Col-0 and *xnd1* mutants was much higher in the absence than in the presence of NaN_3_ (Fig. 1d), suggesting that, with respect to Col-0, loss and gain-of-function *xnd1* mutants show higher and lower plasma membrane aquaporin activity, respectively. Because XND1 serves as a transcription factor, we investigated whether it may alter, either directly or indirectly, the abundance of the corresponding aquaporin transcripts in roots. Thus, we considered all the 13 *Plasma membrane Intrinsic Protein* (*PIP*) genes and investigated the three T-DNA insertion *xnd1* lines and *XND1* overexpressors described above (*35S::XND1*, *XCP2p::GFP–XND1*), taking Col-0 and *XCP2p::GFP* plants as controls. None of the *PIP* genes showed differences in expression that would correlate to *L*p_r_ (Supplementary Figure 10), suggesting that effects of XND1 on aquaporin function are not mediated through *PIP* transcript abundance. The overall data indicate that XND1 acts as a negative regulator of *L*p_r_ by repressing both xylem development and aquaporin activity. The latter effects are not yet understood and may well be indirect.

### XND1 negatively acts on plant drought stress tolerance

**Fig. 6:**
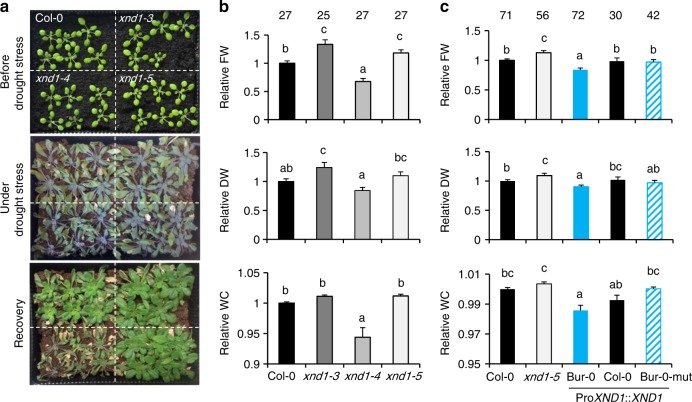
XND1 negatively regulates plant drought stress tolerance. **a** Shoot phenotypes of Col-0 and *xnd1* plants at 22 days postgermination (upper panel), after water deprivation for 24 additional days (middle panel), and at 5 days after rewatering (lower panel). **b** Relative fresh weight (FW), dry weight (DW) and water content (WC) in shoots of indicated genotypes at 5 days after rewatering (see **a**). All data were normalized to values measured in Col-0 plants from the same pot. The figure shows mean values ± SE from the indicated number of plants and four biological replicates. One-way ANOVA (Fisher’s LSD, *P* < 0.05) was used to test the significance of the data. **c** Same parameters as in **b** but for Col-0, *xnd1–5*, and *xnd1–5* plants complemented with the Bur-0, Col-0, or Bur-0-mut *XND1* alleles. Three to five independent transgenic lines were phenotyped for each allele. Mean values ± SE from the indicated number of plants are shown. One-way ANOVA (Fisher’s LSD, *P* < 0.05) was used to test the significance of the data

By determining the efficiency of water supply to the plant’s aerial parts, root hydraulics can play a key role in maintaining the overall plant water status, especially in conditions of strong water demand or water deprivation. To investigate the general role of XND1 in plant water relations, we first compared the wild type (Col-0) and three *xnd1* genotypes grown under various water regimes. Under sufficient water supply, all genotypes showed comparable growth performance, although *xnd1–4* showed a slight deficit in shoot water content (Supplementary Figure 11A, B). When seedlings were grown for three weeks in these favorable conditions and thereafter subjected to a long-term (24 days) water deprivation, all plant genotypes showed signs of severe stress and growth retardation, which, however, were the most pronounced in *xnd1–4* (Fig. 6a). Plant resilience to these extreme conditions was investigated at 5 days after rewatering. With respect to Col-0 plants, loss-of-function *xnd1* mutants showed a higher shoot fresh weight (FW) and a tendency to higher shoot DW (Fig. 6b and Supplementary Figure 11C). The gain-of-function *xnd1* plants (*xnd1–4*) showed a mirror phenotype with reduced shoot growth (lower FW and DW) and reduced shoot water content (Fig. 6b and Supplementary Figure 11C). The overall data indicate that XND1 negatively acts on plant tolerance to water deficit. To address the significance of *XND1* natural variation in plant drought tolerance, we investigated *xnd1–5* lines complemented with the Bur-0, Col-0, or Bur-0 SNP_UTR_-mutated allelic forms of *XND1*. Under water sufficient condition, all complemented lines showed comparable growth performance with respect to Col-0 and *xnd1-5*, with a slight reduction in shoot water content in the Bur-0 allele complemented lines (Supplementary Figure 12). After successive drought and recovery, all complemented lines showed lower shoot FW than *xnd1-5* indicating the functionality of the three *XND1* allelic forms in these conditions (Fig. 6c and Supplementary Figure 13). Interestingly, complementation with the Bur-0 allele resulted in lower shoot FW than with the Col-0 or Bur-0 SNP_UTR_-mutated allele (Fig. 6c and Supplementary Figure 13). In addition, the shoot DW and water content of the Bur-0 allele complemented lines was lower than in lines containing the Col-0 or Bur-0 SNP_UTR_-mutated allele, respectively (Fig. 6c and Supplementary Figure 13). These differences establish that natural variation at *XND1*, at the SNP_UTR_ in particular, confers variations in integrated plant responses to drought.

### XND1 contributes to protection against a vascular pathogen

**Fig. 7:**
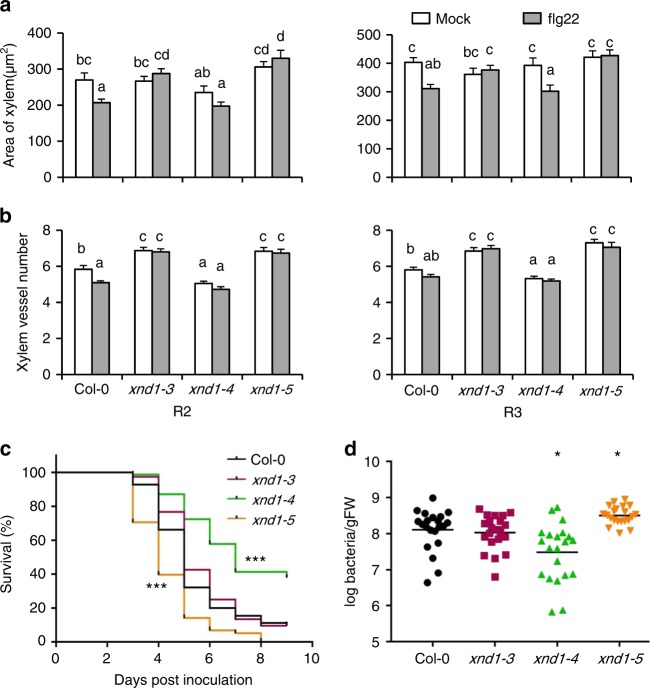
XND1 mediates the inhibition of xylem formation by flg22 and resistance to *R. solanacearum*. **a**, **b** Plants of the indicated genotype were grown for 4 days in the absence (mock) or in the presence of 0.25 µM flg22 (flg22). R2 and R3 root segments (see Fig. 5a) were cross-sectioned and xylem area (**a**) and xylem vessel number (**b**) were measured. Data are means ± SE from 18–50 sections in 6–10 plants and two independent experiments. One-way ANOVA (Fisher’s LSD, *P* < 0.05) was used to test the significance of the data. **c** The indicated genotypes were inoculated with *R. solanacearum* through root dipping and plant survival (in %) was scored at the indicated days postinoculation. Cumulated data from three independent biological experiments, each with three technical repeats comprising at least 24–32 plants. Stars indicate significant differences from Col-0 based on a Gehan–Breslow–Wilcoxon test (*P* < 0.0001). The Col-0 and *xnd1–3* curves were not significantly different (*P* = 0.1304). **d** *in planta* growth measurement of *R. solanacearum* at 3 days after inoculation of the indicated genotypes. Each dot represents a replicate of two plants and the black line indicates the mean of all the replicates. Cumulated data from four independent biological repeats. Asterisks indicate a significant difference with Col-0 (Mann–Whitney test*; P* < 0.05)

Plant vasculature serves as an infection route for pathogens and its development is targeted by multiple biotic factors. Along these lines, abundance of *XND1* transcripts in *Arabidopsis* is enhanced up to fivefold by bacterial infiltration (e.g., *Pseudomonas syringae* pv. *phaseolicola)* and exposure to pathogen-associated molecular patterns (e.g., flg22) (see *Arabidopsis* eFP Browser database at http://bar.utoronto.ca/efp_arabidopsis/cgi-bin/efpWeb.cgi). These data led us to examine in closer details the effects of flg22 on xylem formation. A treatment with 0.25 µM flg22 for 4 days reduced the overall xylem area in R2 and R3 segments of Col-0, but not in *xnd1–3* or *xnd1–5* (Fig. 7a). In R2 root segments of Col-0, xylem vessel number was reduced by flg22 whereas it was unresponsive to the peptide in corresponding root segments of the three *xnd1* insertion mutants (Fig. 7b). Thus, XND1 mediates in part adjustments of vasculature formation under biotic constraints. The significance of these effects was further tested by exposing roots of various *xnd1* genotypes to a soil borne pathogenic bacterium (*Ralstonia solanacearum)* and scoring plant infection symptoms for up to 9 days (Fig. 7c, Supplementary Figure 14A). Based on plant survival rate, the *xnd1–3* knock-down line was indistinguishable from Col-0. In contrast, *xnd1–5* and *xnd1–4* showed, with respect to Col-0, a higher and lower susceptibility to the bacterial strain, respectively. Measurements of bacterial growth in rosettes confirmed this ranking, indicating that at 3 days postinoculation, *xnd1–5* and *xnd1–4* showed *in planta* bacterial multiplication that was higher (*P* = 0.007) and lower (*P* = 0.004), respectively, than in Col-0 (Fig. 7d). At a later stage (4 days postinoculation), all genotypes exhibited similar bacterial contents (Supplementary Figure 14B). Transformation of *xnd1–5* with the Bur-0, Col-0, or Bur-0 SNP_UTR_-mutated allelic forms of *XND1* abolished the higher susceptibility of *xnd1–5* to *R*. *solanacearum*, with bacterial wilt phenotypes of the complemented lines similar to that of Col-0 (Supplementary Figure 14C). The overall data indicate that XND1 limits bacterial proliferation in the plant and subsequent bacterial wilt appearance, supporting the idea that reduced xylem formation contributes to plant protection against bacterial infection.

## Discussion

Recent work from our group^19^ has shown that, although technically challenging and under strong environmental and developmental dependency, root hydraulics (*L*p_r_) is amenable to quantitative genetics analyses. With respect to the recombinant population mapping approach used in our previous work, GWA studies could be more straightforward for identifying candidate genes quantitatively acting on a trait of interest. In any case, it is described that these two approaches are certainly complementary to reveal the genetic architecture of a trait^25^. In the present study, a one shot phenotyping of 143 accessions allowed identification of two novel genes, *At1g36240* and *At5g64530*, which proved to act as negative regulators of *L*p_r_.

While additional work is required to assess the role of At1g36240, several complementary lines of evidence indicate that At5g64530 (*XND1*) corresponds to the gene spotted by the GWA signal on Chr 5. First, loss- and gain-of-function of *XND1* in Col-0 insertion mutants resulted in opposite *L*p_r_ phenotypes. Although very encouraging to confirm our candidate gene’s association^26^, this is not sufficient per se. Thus, we also showed that four natural *XND1* allelic forms have distinct abilities to complement the enhanced *L*p_r_ phenotype of *xnd1–5* knock-out plants. Remarkably, the corresponding haplogroups, each containing 7–38 natural accessions, showed consistent quantitative differences in *L*p_r_ further supporting the idea that an allelic series at *XND1* significantly modulates root hydraulics at the species-wide level. Additionally, an epistatic *L*p_r_ QTL corresponding to the *XND1* region was mapped in a Bur-0 × Col-0 RIL population, with effects on *L*p_r_ consistent with those of corresponding *XND1* alleles. Because of confounding genetic relatedness or allelic heterogeneities, the statistics of GWAS detection can lead to complex patterns where causal polymorphisms in the gene of interest are away from and do not show LD with the most significantly associated SNPs^27^. The present work may typically represent such case since the Chr 5 GWAS peak SNP (SNP_peak_) was not in strong LD with any single SNP of neighboring genes in a 48 kb region (Supplementary Figure 2). Yet, a gene-based association study with a different accession set and an extended polymorphism data, pointed to a SNP present in the 5′-UTR of *XND1* (SNP_UTR_) as two allelic forms, and located at a 7901 bp distance from the Chr 5 GWAS peak SNP. The fact that SNP_UTR_ and SNP_peak_ do not appear in strong LD (*r*^2^ = 0.23, Supplementary Figure 2), could be explained in part by a bias due to the relatively low minor allele frequency (14%) at SNP_UTR_. Again, we used transgenic complementation to assess the functional significance of this variation: the contribution of this SNP_UTR_ to *L*p_r_ was established by site-directed allelic conversion of a Bur-0 allele and functional expression in a Col-0 background. Thus, the hyperfunctionality of the Bur-0 vs. Col-0 allele is due in part to a T-to-C substitution at this position. The failure of a symmetrical mutation in the Col-0 allele to modify *L*p_r_ indicates that SNP_UTR_ functionally interacts with other intragenic components to yield the *XND1*-dependent variation of *L*p_r_. In particular, SNPs in the 3’UTR were not considered and could contribute to natural phenotypic variation. In summary, the overall work provides a thorough dissection of natural variations at *XND1* and demonstrates their contribution to root hydraulics.

Causal polymorphisms can be located in coding regions, in regulatory non-coding regions (this work) or can correspond to genomic structural variations^28^. Their identification usually provides highly relevant insights into the function and regulation of the gene or gene product of interest. In the case of XND1, two protein interaction motifs present in the C-terminal domain are essential for its function as they allow binding to the general cell cycle and differentiation regulator RETINOBLASTOMA-RELATED^29^. Yet, these motifs were highly conserved in the accession panel of this study and seem not to underlie *XND1*-based natural variation of *L*p_r_.

Here, we found that *L*p_r_ natural variation was not correlated to *XND1* transcript abundance when natural accessions were assembled in functionally discriminating haplogroups (Fig. 4b, c; Supplementary Figure 15). Knowing that the 5′-UTR of plant genes can harbor regulatory elements that affect RNA structure, RNA–protein interactions and/or ribosome recruitment^30,31^, we investigated whether allelic variation at SNP_UTR_ interferes with XND1 translation efficiency. Because XND1 is a transcription factor that is lowly abundant and primarily confined to the nucleus of xylem cells, we monitored fluorescence of a XND1–GFP construct in a specific root tip region as it was the most suitable to proper quantification. Although a definitive analysis such as ribosome profiling remains to be done, our results based on ratio of protein and mRNA abundances in cells ectopically expressing *XND1* suggest that SNP_UTR_ potentially affects translational efficiency. As a result, we propose that variation at SNP_UTR_ leads to distinct XND1 protein abundance, xylem differentiation and *L*p_r_. We also stress that, as indicated by the local association study, SNP_UTR_ is likely not the only causal SNP, and other variants, for instance specific to haplotype H6 (Fig. 2c), may also be functional.

The identification of *XND1* in our GWA study was strikingly in line with the notion that NAC transcription factors control water-conducting structures from primitive terrestrial plants to most recently evolved vascular plants^15,22,32^. Further, XND1 is an angiosperm-specific NAC that contributes to specific vessel differentiation features in these plants^29^. Consistent with earlier studies^22^, we validated that XND1 negatively acts on xylem differentiation and, based on Hagen–Poiseuille law^33^, we propose that its developmental function results in an axial hydraulic limitation in root tips. A genetic link between xylem function and root hydraulics has previously been pointed by Lefebvre et al.^18^, based on the dramatic drop in *L*p_r_ displayed by *esk1* loss-of-function mutants. With respect to the general collapse of xylem vessels and pleiotropic stress phenotypes shown by these mutants, *xnd1* genotypes showed milder alterations of xylem tissues, specifically in roots tips. Thus, a slightly altered abundance and overall size of the xylem vessels seem to be sufficient to perturb *L*p_r_. A more precise analysis indicated, however, that XND1 may also act on aquaporin function to reduce *L*p_r_. This could be due to indirect effects on aquaporin expression and/or function due to locally altered vascular tissue differentiation. While these conclusions rely on analysis of *xnd1* transgenic lines, the precise mechanisms that underlie effects of natural *XND1* variants on *L*p_r_ remain to be explored.

Beyond root hydraulics, our study underscores a general role of xylem in plant response to environmental stresses. In particular, we showed that XND1 negatively acts on drought tolerance. Based on the phenotype of *XND1* overexpressors, we propose that reduced xylem differentiation and water transport in root tips hampers water extraction in deep, newly colonized soil strata. A reduced leaf water content was also observed in well-watered conditions, suggesting a general hydraulic limitation in these plants. These findings contribute to a longstanding debate on the role of xylem in drought tolerance. Genetic selection programs in wheat and rice have targeted xylem morphology using root thickness as a proxy^7,34^. However, the significance of these variations with respect to drought adaptation has been controversial (discussed by Lynch et al.^5^). In wheat, reduced xylem diameter was linked to better growth in dry environments whereas, in rice, genotypic variation in xylem vessel diameter was not linked to the plant’s capacity to regulate leaf water status under drought^11^. The genetic variants of *XND1* investigated in the present work provide more precise insights into these questions, at least in the case of *Arabidopsis*. We also realize that, besides efficiency for conducting the transpiration stream, xylem anatomy contributes to cavitation resistance and thereby to tolerance to extreme drought events. However, this critical trait, which shows high phenotypic and genotypic plasticity in trees^35^, could not be addressed in our work since no natural cavitation has ever been described in *Arabidopsis*.

Xylem differentiation is sensitive to many more environmental factors than water availability^36,37^. For instance, a link between PAMP-triggered immunity and xylem differentiation pathways involving plant glycogen synthase kinase 3 proteins is emerging^38,39^. In the present work, we investigated the significance of *XND1* induction by flg22, and found this gene to mediate the inhibiting effects of the elicitor on vascular formation. A general role for *XND1* in plant defense was further investigated using *R. solanacearum* a prototypal root vascular pathogen that causes bacterial wilt in many plant species^40^. As for drought assays, series of hypo- and hyperfunctional, artificial and natural alleles were used to address *XND1* function. Consistent with its induction upon infection, enhanced expression of *XND1* in *xnd1–4* led to plants more tolerant to *R. solanacearum* than Col-0, whereas a loss-of-function mutation (*xnd1-5*) caused a mirror phenotype that could be complemented using either Col-0 or Bur-0 alleles. Several modes of actions may explain the role of XND1 in the present pathosystem. In agreement with *in planta* bacterial growth measurements, we propose that restriction or delayed differentiation of the vasculature mediated by XND1 may antagonize microbe propagation, and/or the establishment of an aqueous living space for pathogens^41^, thereby contributing to plant defense. Alternatively, and as shown for other NACs^42^, XND1-mediated transcriptional reprogramming could enhance plant immunity.

Because of their general role in differentiation of water-conducting tissues, NACs have played a central role during plant evolution, and contributed to their adaptation to aerial environments^15^. Our study shows that natural selection is still at work in higher plants, and refines the role of a crucial NAC member in diverse vascular functions. By negatively acting on the differentiation of xylem vessels in root tips, XND1 sensitizes the plant to extreme drought events while directly or indirectly providing a partial protection against systemic pathogen attack. Thus, XND1 mediates a trade-off between plant responses to biotic and abiotic stresses. While environmental stresses can occur independently, sequentially or in combination, this work exclusively reports on effects of individual stresses. Since the role of *XND1* could conceivably be more complex when stresses occur in concert than when applied individually, additional dimensions of the role of this gene in plant stress responses remain to be explored. Nevertheless, our work indicates that natural variation at *XND1* may contribute to the extensive geographic distribution of species such as *Arabidopsis*^43^. Interestingly, the minor allelic form (14% of the 112 accessions) carrying SNP_UTR_-C was associated with hyperfunctional alleles, lower *L*p_r_ and lower drought tolerance. This indicates that the selective balance of drought over pathogen attacks may have been predominant to maintain SNP_UTR_-T as the major allele. While our study emphasizes the adaptive significance of *XND1* variations in natural plant populations, it will also be crucial to investigate the genetic variation of this gene and of other NAC homologs in trees^32^ and herbaceous crops, for applied purposes such as wood quality, productivity under drought conditions or disease control.

## Methods

### Plant materials

Seeds for natural accessions of *Arabidopsis thaliana* (Supplementary Data 1) or T-DNA insertion lines (Supplementary Table 1) were obtained from the Nottingham Arabidopsis Stock Centre (NASC) except for seeds of FLAG_153A06 which came from the Versailles Arabidopsis Stock Center. When required, T-DNA insertions were confirmed by PCR using primers listed in Supplementary Table 2. *XND1* overexpression lines under control of a *CaMV35S* or xylem specific *XCP2* promoter^44^ were as described previously^22,29^.

### Hydroponic growth conditions

Seeds were surface sterilized and sown on petri plates (12 × 12 cm, Gosselin, BP124-04) with 0.5 × Murashige and Skoog (MS) solid medium [2.2 g L^−1^ MS (Sigma, M5519), 10 g L^−1^ sucrose (Euromedex, 200–301 B), 0.5 g L^−1^ MES (Euromedex, EU0033), and 0.7% agar (Sigma, A4675), pH 5.7 adjusted using KOH]. Following stratification for 2 days in the dark at 4 °C, plates were incubated vertically for 9 days in a growth chamber at 20 °C and 70% relative humidity, with cycles of 16 h of light (250 µmol photons m^−2^ s^−1^) and 8 h of night. Plants were then transferred in hydroponic culture under the same growth conditions. The hydroponic medium (0.75 mM MgSO_4_, 0.5 mM KH_2_PO_4_, 1.25 mM KNO_3_, 1.5 mM Ca(NO_3_)_2_, 50 µM Fe-EDTA, 100 µM Na_2_SiO_3_, 50 µM H_3_BO_3_, 12 µM MnSO_4_, 1 µM ZnSO_4_, 0.7 µM CuSO_4_, 0.24 µM Na_2_MoO_4_) was replaced weekly.

### Root hydraulic conductivity (*L*p_r_) measurements

Measurements were performed essentially as described^3^ on 21- to 23-day-old plants cultured in hydroponics. The intact root system of a freshly de-topped plant was inserted into a pressure chamber filled with hydroponic solution (pH 6.5 adjusted using KOH), and sealed with a silicon dental paste (PRESIDENT Light Body, Coltene, Switzerland). The root system was subjected to a pressure pretreatment at 350 kPa for 10 min to attain equilibration of sap flow exuded from the hypocotyl section, and to successive treatments at 320, 160, and 240 kPa, each for 2 min. High-accuracy flow meters (Bronkhorst, France) in combination with a LabVIEW-derived application were used to record the rate of pressure (*P*)-induced sap flow (*J*_v_). DW_r_ was determined after desiccation at 80 °C for at least 24 h. *L*p_r_ (in ml g^−1^ h^−1^ MPa^−1^) was calculated as: *L*p_r_ = *J*_v_/(DW_r_ ∙ *P*). In azide (NaN_3_) inhibition experiments, *J*_v_ was measured at continuous 320 kPa and its percentage inhibition was measured at 30 min following the addition of 1 mM NaN_3_.

### GWA, candidate-based association and haplotype analysis

For GWA analysis, *L*p_r_ was measured on 143 natural accessions, each with 6 individual plants randomized over a measuring period of four weeks (Supplementary Data 1). For the calculation of broad-sense heritability, average intra-accession variance was taken as the environmental variance, and data for all individuals were used for calculating phenotypic variance. A conditional GWA analysis was conducted on mean *L*p_r_ values using the GWAPP web interface (https://gwas.gmi.oeaw.ac.at/) and the accelerated mixed-model (AMM) algorithm method with cofactors^21^. Genotype data for 206,087 SNPs from 250k SNP chip^20^ were used to carry out GWA analysis, and only SNPs with MAF > 0.05 (equivalent to a minor allele count (MAC) ≥ 8) were considered. For conditional analysis, four successive steps were performed following an initial calculation with AMM. At each step, one among the nine most highly associated SNPs identified in the previous calculation was arbitrarily selected as a cofactor. In practice, four SNPs (Chr1-P13,612,169; Chr 5-P25,787,448; Chr 5-P21,846,701, and Chr4-P17,518,747) were stepwise included as cofactors. To correct for multiple testing, a Bonferroni correction with a nominal significance threshold (*α*) of 0.05 was applied, corresponding to an uncorrected *P* value of 2.61 × 10^−7^. The proportion of genetic variance explained by SNP (13,612,169/Chr1) and SNP (25,787,448/Chr 5) was determined using coefficients of determination from simple linear regressions. For *XND1*-based local association analysis, we extracted from Salk Arabidopsis 1001 Genomes database (http://signal.salk.edu/atg1001/index.php) genomic sequences of *XND1* (encompassing a region from 2 kb upstream to 300 bp downstream of *XND1* coding sequence) from 112 accessions. Of these, 85 belong to the initially investigated panel for GWA mapping (Supplementary Data 2). Association analysis between polymorphic sites (including INDELs and SNPs with MAF > 0.05) and *L*p_r_ were performed with TASSEL version 5 using a generalized linear model^45^. The significance threshold was set at a *P* value of 0.05 per marker number. Haplotypes were classified based on eight polymorphic sites showing the lowest *P* values according to *XND1*-based local association analysis. The haplogroups containing at least five accessions were used for further comparative analysis.

### Genetic complementation of *xnd1*

A 7088 bp genomic region harboring *XND1* was amplified from Col-0, Bur-0, Ty-0, and Fei-0 genomic DNA using a iProof High-Fidelity PCR Kit (Bio-Rad) and cloned into a pGreen0179 vector^46^. A QuikChange Site-Directed Mutagenesis Kit (Agilent stratagene) was used to mutate the SNP_UTR_ (Chr5_P25,795,349) of the cloned Col-0 or Bur-0 *XND1* fragments. All primer sequences used are listed in Supplementary Table 2. The constructs were confirmed by sequencing and transferred into *xnd1–5* mutant plants using the floral dip method^47^. For each construct, three to five independent homozygous transgenic lines were selected in T3 generation on 30 mg L^−1^ hygromycin B (Sigma), checked for *XND1* expression (see below) and phenotyped for *L*p_r_.

### Quantitative gene expression

*XND1* and *PIP* mRNA abundance was characterized in transgenic lines and/or natural accessions using qRT-PCR. Total RNA was extracted from *Arabidopsis* roots and reverse-transcribed using a SV Total RNA Isolation System (Promega) and M-MLV reverse transcriptase (Promega), respectively. PCR was performed on an optical 384-well plate with a LightCycler^®^ 480 system (Roche) using SYBR Green I Master (Roche) or SYBR Premix Ex Taq (TaKaRa), according to the manufacturer’s instructions. *TIP41-like protein* (At4g34270), *PP2A3* (At1g13320), and *SAND family protein* (At2g28390) were selected as reference genes^48^, based on their expression stability among accessions evaluated using a NormFinder software^49^. All primer sequences used are listed in Supplementary Table 2. Relative expression levels were determined using the 2^(−ΔΔC(T))^ method^50^, and calibrated with respect to transcript abundance in the wild-type control, unless otherwise stated.

### Characterization of RSA

Roots of hydroponically grown 22-day-old plants were harvested and preserved in 20% ethanol solution. The whole root systems were immersed in water and positioned on a square petri dish (24 cm × 24 cm), so as to avoid root overlapping, and then imaged using an Epson V850 Pro scanner at 600 dpi. Primary and total root lengths, and lateral root density were analyzed with an OPTIMAS software (version 6.1). The lateral root density was determined on the primary root, in a 12 cm region starting from the tip. Root DW was determined after desiccation at 80 °C for at least 24 h.

### Root histological analyses

Roots of 21- to 23-day-old plants were cut in 2-cm-long segments starting from 0.3 cm of the tip. Root segments were embedded in 4% low-melting point agarose (Euromedex, 1670-B) and cross-sectioned (~100 μm thickness) using a Micro-Cut H1200 Vibratome (Bio-Rad) according to the manufacturer’s instruction. Xylem morphology was observed under a BH-2 Bright field Microscope (Olympus) and quantified using ImageJ. Xylem vessels were identified from their thickened cell wall. Xylem size and abundance were assessed from the area of all vessels and vessel number, respectively. For testing the effect of flg22 on xylem formation, 19-day-old plants were exposed for 4 days to a hydroponic solution containing 0.25 µM flg22 or 0.025% DMSO as mock treatment, and xylem morphology was analyzed as described above.

### Expression of fluorescent XND1 fusion proteins

A 1465 bp region harboring *XND1* 5′-UTR and coding sequence, without stop codon and in its wild-type or SNP_UTR_-mutated Bur-0 form, was amplified from above mentioned *XND1*-pGreen0179 clones. The fragment was cloned using the Gateway Technology (Invitrogen), downstream of a *35SCaMV* promoter and in fusion with *GFP* in a pGWB505 vector^51^. The primers used are listed in Supplementary Table 2. The constructs were confirmed by sequencing and transferred into *xnd1–5* mutant plants. For each construct, nine to ten independent transgenic lines were selected in T2 generation on 30 mg L^−1^ hygromycin B (Sigma), cultured in hydoponics and checked for *XND1-GFP* transcript abundance and GFP fluorescence intensity in roots. The primers used for qRT-PCR are listed in Supplementary Table 2. For quantification of the GFP fluorescence intensity, roots of 21- to 23-day-old plants were observed using a fluorescence microscope (Zeiss Axio Observer 7) and mean gray values in 400 µm root tips were quantified using an ImageJ program (NIH, USA). Data were normalized to the corresponding value of the line C9-3, which possessed the lowest transcript abundance and fluorescence intensity.

### Drought stress treatments

Col-0 plants, *xnd1* T-DNA insertion and complementation lines were grown in trays filled with peat soil (Neuhaus Humin Substrat N2, Klasmann-Deilmann). Four to six trays were used per experiment. Each tray was divided in four quarters, each containing six plants of a specific genotype (quarter-split manner). The dimensions (length × width × depth) of the trays were 18 cm × 13 cm × 5.5 cm. Plants were maintained in a growth chamber at 20 °C and 65% relative humidity, with cycles of 8 h of light (250 µmol photons m^−2^ s^−1^) and 16 h of night, and sufficient watering. After 22 days, plants were subjected to drought by withholding water for 24 days and re-irrigated for 5 days. Gravimetric soil water content was around 30% at the end of the water deficit period. The control treatment was conducted in the same conditions, but with continuous watering. Shoot fresh weight (FW) was determined immediately after harvest whereas shoot DW was measured after further desiccation for at least 4 days at 60 °C. Shoot water content was calculated as the FW-to-DW difference. All data were normalized to the corresponding mean value of Col-0 plants in the same tray.

### Bacterial inoculations

Seeds of Col-0 plants and *xnd1* T-DNA insertion lines were surface sterilized for 20 min with a 12% sodium hypochlorite solution, washed five times with sterile water and sown on a MS solid medium. After 8 days at 20 °C in a growth chamber, plantlets were transferred to Jiffy pots (Jiffy France, Lyon, France) and grown for 3 weeks under short days conditions at 22 °C and 70% relative humidity with 9 h of light (250 μmol m^−2^ s^−1^). Exposed roots of the plants were immersed for 20 min in a suspension containing 10^8^ bacteria/mL of *R. solanacearum* GMI1000 strain^52^. Inoculated plants were then transferred to a new tray on a firm surface of potting soil, and incubated in a growth chamber at 75% relative humidity with cycles of 12 h of light (100 μmol m^−2^ s^−1^) at 27 °C and 12 h of night at 26 °C. Plant position was randomized prior to inoculation. Symptom appearance was scored daily and independently for each plant, using a macroscopic scale describing the observed wilting: 0, no wilting; 1, 25% of leaves wilted; 2, 50%; 3, 75%; 4, complete wilting. For subsequent analysis, the data were transformed into a binary index: 0, < 50% leaves wilted; 1, ≥ 50% wilted leaves in order to construct survival curves. We then applied the Kaplan–Meier survival analysis^53^ with the Gehan–Breslow–Wilcoxon method to compute the *P* value and test the null hypothesis of identical survival experience of the tested mutant. A *P* value lower than 0.05 was considered to be significant. The survival curves represent a pool of three technical replicates, each with 24–32 plants, and 3 independent biological replicates corresponding to 240 plants for Col-0 and for each of the *xnd1* T-DNA insertion lines. For bacterial internal growth measurements, a *R. solanacearum* GMI1000 derivative strain carrying a gentamycine resistance cassette^54^ was used. Rosettes of three to six pairs of plants were harvested at 3 and 4 days postinoculation, sterilized in 70% ethanol and rinsed three times in sterile water. The rosettes were then weighted, grinded and re-suspended in sterile water. Bacterial concentrations were determined by plating dilutions on B medium. Four biological replicates were done. Comparison of *in planta* bacterial multiplication in *xnd1* genotypes with that in Col-0 was performed through a Mann–Whitney test. All statistical analyses were performed with a Prism version 5.0 software (GraphPad Software, San Diego, CA, USA).

### Statistical analyses

Unless otherwise indicated, statistical significance of the data was assessed using either a Student’s *t* test (^*^P < 0.05) or one-way ANOVA (lowercase letters: *P* < 0.05). Student’s *t* tests were performed using EXCEL whereas a STATISTICA software was used for ANOVA and multiple comparison tests (Fisher’s least significant difference).

## Electronic supplementary material

Supplementary Information

Description of Additional Supplementary Files

Supplementary Data 1

Supplementary Data 2

Supplementary Data 3

Supplementary Data 4

## Data Availability

The data supporting the findings of the study are available as Supplementary data or from the corresponding author on reasonable request.

## References

[1] Maurel C (2015). Aquaporins in plants. Physiol. Rev..

[2] Shahzad Z, Amtmann A (2017). Food for thought: how nutrients regulate root system architecture. Curr. Opin. Plant. Biol..

[3] Sutka M (2011). Natural variation of root hydraulics in *Arabidopsis* grown in normal and salt stress conditions. Plant Physiol..

[4] Adachi S (2010). Characterization of a rice variety with high hydraulic conductance and identification of the chromosome region responsible using chromosome segment substitution lines. Ann. Bot..

[5] Lynch JP, Chimungu JG, Brown KM (2014). Root anatomical phenes associated with water acquisition from drying soil: targets for crop improvement. J. Exp. Bot..

[6] Uga Y (2013). Control of root system architecture by DEEPER ROOTING 1 increases rice yield under drought conditions. Nat. Genet..

[7] Uga Y, Okuno K, Yano M (2008). QTL underlying natural variation in stele and xylem structures of rice root. Breed. Sci..

[8] Vadez V (2014). Root hydraulics: the forgotten side of roots in drought adaptation. Field Crops Res..

[9] Steudle E, Peterson CA (1998). How does water get through roots?. J. Exp. Bot..

[10] Choat B (2012). Global convergence in the vulnerability of forests to drought. Nature.

[11] YAMBAO ELIZABETH B., INGRAM KEITH T., REAL JOSELITO G. (1992). Root Xylem Influence on the Water Relations and Drought Resistance of Rice. Journal of Experimental Botany.

[12] De Rybel B, Mahonen AP, Helariutta Y, Weijers D (2016). Plant vascular development: from early specification to differentiation. Nat. Rev. Mol. Cell Biol..

[13] Olsen AN, Ernst HA, Leggio LL, Skriver K (2005). NAC transcription factors: structurally distinct, functionally diverse. Trends Plant. Sci..

[14] Heo JO, Blob B, Helariutta Y (2017). Differentiation of conductive cells: a matter of life and death. Curr. Opin. Plant Biol..

[15] Xu B (2014). Contribution of NAC transcription factors to plant adaptation to land. Science.

[16] Pfister A (2014). A receptor-like kinase mutant with absent endodermal diffusion barrier displays selective nutrient homeostasis defects. eLife.

[17] Ranathunge K, Schreiber L (2011). Water and solute permeabilities of *Arabidopsis* roots in relation to the amount and composition of aliphatic suberin. J. Exp. Bot..

[18] Lefebvre V (2011). ESKIMO1 disruption in *Arabidopsis* alters vascular tissue and impairs water transport. PLoS One.

[19] Shahzad Z (2016). A potassium-dependent oxygen sensing pathway regulates plant root hydraulics. Cell.

[20] Horton MW (2012). Genome-wide patterns of genetic variation in worldwide *Arabidopsis thaliana* accessions from the RegMap panel. Nat. Genet..

[21] Seren U (2012). GWAPP: a web application for genome-wide association mapping in *Arabidopsis*. Plant Cell.

[22] Zhao C, Avci U, Grant EH, Haigler CH, Beers EP (2008). XND1, a member of the NAC domain family in *Arabidopsis thaliana*, negatively regulates lignocellulose synthesis and programmed cell death in xylem. Plant J..

[23] Zhao C, Craig JC, Petzold HE, Dickerman AW, Beers EP (2005). The xylem and phloem transcriptomes from secondary tissues of the *Arabidopsis* root-hypocotyl. Plant Physiol..

[24] The 1001 Genomes Consortium. (2016). 1135 genomes reveal the global pattern of polymorphism in *Arabidopsis thaliana*. Cell.

[25] Bazakos C, Hanemian M, Trontin C, Jimenez-Gomez JM, Loudet O (2017). New strategies and tools in quantitative genetics: how to go from the phenotype to the genotype. Annu. Rev. Pant Biol..

[26] Verslues PE, Lasky JR, Juenger TE, Liu TW, Kumar MN (2014). Genome-wide association mapping combined with reverse genetics identifies new effectors of low water potential-induced proline accumulation in *Arabidopsis*. Plant Physiol..

[27] Kerdaffrec E (2016). Multiple alleles at a single locus control seed dormancy in Swedish *Arabidopsis*. eLife.

[28] Grimm DG (2017). easyGWAS: A cloud-based platform for comparing the results of genome-wide association studies. Plant Cell.

[29] Zhao C (2017). XYLEM NAC DOMAIN 1, an angiosperm NAC transcription factor, inhibits xylem differentiation through conserved motifs that interact with RETINOBLASTOMA-RELATED. New Phytol..

[30] Hinnebusch AG, Ivanov IP, Sonenberg N (2016). Translational control by 5’-untranslated regions of eukaryotic mRNAs. Science.

[31] Merchante C, Stepanova AN, Alonso JM (2017). Translation regulation in plants: an interesting past, an exciting present and a promising future. Plant J..

[32] Grant EH, Fujino T, Beers EP, Brunner AM (2010). Characterization of NAC domain transcription factors implicated in control of vascular cell differentiation in *Arabidopsis* and Populus. Planta.

[33] Tixier A (2013). *Arabidopsis thaliana* as a model species for xylem hydraulics: does size matter?. J. Exp. Bot..

[34] Richards RA, Passioura JB (1989). A breeding program to reduce the diameter of the major xylem vessel in the seminal roots of wheat and its effect on grain yield in rain-fed environments. Aust. J. Agric. Res..

[35] Wortemann R (2011). Genotypic variability and phenotypic plasticity of cavitation resistance in *Fagus sylvatica* L. across Europe. Tree Physiol..

[36] Plavcova L, Hacke UG (2012). Phenotypic and developmental plasticity of xylem in hybrid poplar saplings subjected to experimental drought, nitrogen fertilization, and shading. J. Exp. Bot..

[37] Atkinson CJ, Taylor JM (1996). Effects of elevated CO_2_ on stem growth, vessel area and hydraulic conductivity of oak and cherry seedlings. New Phytol..

[38] Kondo Y (2014). Plant GSK3 proteins regulate xylem cell differentiation downstream of TDIF-TDR signalling. Nat. Commun..

[39] Kang S (2015). The *Arabidopsis* transcription factor BRASSINOSTEROID INSENSITIVE1-ETHYL METHANESULFONATE-SUPPRESSOR1 is a direct substrate of MITOGEN-ACTIVATED PROTEIN KINASE6 and regulates immunity. Plant Physiol..

[40] Hayward AC (1991). Biology and epidemiology of bacterial wilt caused by pseudomonas solanacearum. Annu. Rev. Phytopathol..

[41] Xin XF (2016). Bacteria establish an aqueous living space in plants crucial for virulence. Nature.

[42] Nuruzzaman M, Sharoni AM, Kikuchi S (2013). Roles of NAC transcription factors in the regulation of biotic and abiotic stress responses in plants. Front. Microbiol..

[43] Smakowska E, Kong J, Busch W, Belkhadir Y (2016). Organ-specific regulation of growth-defense tradeoffs by plants. Curr. Opin. Plant Biol..

[44] Funk V, Kositsup B, Zhao C, Beers EP (2002). The *Arabidopsis* xylem peptidase XCP1 is a tracheary element vacuolar protein that may be a papain ortholog. Plant Physiol..

[45] Bradbury PJ (2007). TASSEL: software for association mapping of complex traits in diverse samples. Bioinformatics.

[46] Hellens RP, Edwards EA, Leyland NR, Bean S, Mullineaux PM (2000). pGreen: a versatile and flexible binary Ti vector for *Agrobacterium*-mediated plant transformation. Plant Mol. Biol..

[47] Clough SJ, Bent AF (1998). Floral dip: a simplified method for *Agrobacterium*-mediated transformation of *Arabidopsis thaliana*. Plant J..

[48] Czechowski T, Stitt M, Altmann T, Udvardi MK, Scheible WR (2005). Genome-wide identification and testing of superior reference genes for transcript normalization in *Arabidopsis*. Plant Physiol..

[49] Andersen CL, Jensen JL, Orntoft TF (2004). Normalization of real-time quantitative reverse transcription-PCR data: a model-based variance estimation approach to identify genes suited for normalization, applied to bladder and colon cancer data sets. Cancer Res..

[50] Livak KJ, Schmittgen TD (2001). Analysis of relative gene expression data using real-time quantitative PCR and the 2(-Delta Delta C(T)) Method. Methods.

[51] Nakagawa T (2007). Improved Gateway binary vectors: high-performance vectors for creation of fusion constructs in transgenic analysis of plants. Biosci. Biotechnol. Biochem..

[52] Salanoubat M (2002). Genome sequence of the plant pathogen *Ralstonia solanacearum*. Nature.

[53] Bland JM, Altman DG (1998). Survival probabilities (the Kaplan–Meier method). Br. Med. J..

[54] Guidot A (2014). Multihost experimental evolution of the pathogen *Ralstonia solanacearum* unveils genes involved in adaptation to plants. Mol. Biol. Evol..

