# Supplementary material for: Natural variation at XND1 impacts root hydraulics and trade-off for stress responses in Arabidopsis
Source: Nat Commun. 2018 Sep 24;9:3884. doi: 10.1038/s41467-018-06430-8 (PMC6155316; doi:10.1038/s41467-018-06430-8)
Supplement: Supplementary file 1 — Supplementary Information [file 41467_2018_6430_MOESM1_ESM.pdf]

Supplementary Information

Natural variation at *XND1* impacts root hydraulics and trade-off for stress responses in *Arabidopsis*

Tang *et al.*

Supplementary Table 1. *L*p_r characterization of T-DNA mutants of candidate genes.

Locus	Annotation	Stock name	*L*p_r difference compared to WT (%)	n	P value (T-test)
At1g36225	Transposable element	SALK_205307C	8.4	15	0.3366
		WiscDsLoxHs073_12D	-1.6	15	0.8019
At1g36230	Hypothetical protein	GABI_914B01	-6.7	19	0.0990
At1g36240	Ribosomal protein L7Ae/L30e/S12e/Gadd45	SALK_209120C	10.6	38	0.0395
	family protein	SALK_076955	16.9	30	0.0352
At1g36250	Transposable element	SALK_034537C	4.0	14	0.7012
At5g64500	Major facilitator	SALK_093140C	1.4	7	0.8630
	superfamily protein	GABI_338E03	2.4	13	0.3527
At5g64510	Tunicamycin Induced 1, a plant-specific ER	SALK_006509C	-8.2	17	0.3150
	stress-inducible protein	GABI_123G05	13.7	13	0.1623
At5g64520	X-RAY REPAIR CROSS COMPLEMENTING 2 family protein involved in	SALK_029106	-3.1	15	0.6869
	DNA repair	FLAG_153A06	-3.0	12	0.8305
At5g64530	Xylem NAC domain 1	SALK_046891 (*xnd1-3*)	19.9	27	0.0055
	(XND1)	SALK_023898 (*xnd1-4*)	-18.6	38	0.0014
		GABI_162D11 (*xnd1-5*)	29.7	21	4.96E-05

Supplementary Table 2. Nucleotide sequence of primers used for genotyping and complementation of T-DNA insertion lines, site-directed mutagenesis, and qRT-PCR.

Primer name	Sequence (5' to 3')	Use
SALK_046891 (*xnd1-3*)-LP	ACGTACATGCTTTGGTGAGTG	Genotyping
SALK_046891 (*xnd1-3*)-RP	ACATCGATTACTCATTCCCCC	
SALK_023898 (*xnd1-4*)-LP	ACAATTTGGAAGGGGAAAGTG	
SALK_023898 (*xnd1-4*)-RP	TGTATATACCGGGAAGGTCCC	
GABI_162D11 (*xnd1-5*)-LP	TGGTTTTCCTCCTAACCTTAGG	
GABI_162D11 (*xnd1-5*)-RP	GAAGGGAGGCAATGGTACTTC	
SALK_029106-LP	TTTACATCTGGCGATTTTTGC	
SALK_029106-RP	ATCATCATTGGCATTGGAGAC	
FLAG_153A06-LP	GAGTGGCGCTACTATTGATGC	
FLAG_153A06-RP	CTGCATGAGATGATGAAGCAG	
GABI_123G05-LP	CTCAACACCACGTCACACATC	
GABI_123G05-RP	TTCTTCAAAACACCAGCATCC	
GABI_338E03-LP	CATCTGGAGCAAAACCTGAAG	
GABI_338E03-RP	CTGGCAGCGGAATACAGTAAG	
SALK_076955-LP	ACCCTCACCGATTTCTGATTC	
SALK_076955-RP	GCAGGCAGTACCAAGATCAAC	
GABI_914B01-LP	GCCATGATGACGAAAAGAAAG	
GABI_914B01-RP	ATCTTTTGATAGACCCGCCTC	
SALK_LBb1.3	ATTTTGCCGATTTCGGAAC	
GABI_o8409	ATATTGACCATCATACTCATTGC	
FLAG_LB4	CGTGTGCCAGGTGCCCACGGAATAGT	
XND1_full_length-F-Kpn1	ataggtaccAGATTTCATTTCAGGTATTCTTCAA	Complementation
XND1_full_length-R-BamH1	ataggatccATGAGAAGTGTAGTAGGCTGCAA	
XND1-Bur-0-mut-F	GTAGTGAGATCAATATCGTTAGCAATG	Site-directed mutagenesis
XND1-Bur-0-mut-R	CATTGCTAACGATATTGATCTCACTAC	
XND1-Col-0-mut-F	GTAGTGAGATCAACATCGTTAGCAATG	
XND1-Col-0-mut-R	CATTGCTAACGATGTTGATCTCACTAC	
XND1-GFP-F-attB1	ggggacaagtttgtacaaaaaagcaggctACCTCAATCT CTTTCACACAC	Expression of XND-GFP fusion protein
XND1-GFP-R-attB2	gggaccactttgtacaagaaagctgggtACGGTAAGCT TACTTCGTCAAG	
TIP41(At4g34270)-qPCR-F	GTGAAAACTGTTGGAGAGAAGCAA	qRT-PCR
TIP41(At4g34270)-qPCR-R	TCAACTGGATACCCTTTCGCA	
PP2A3(At1g13320)-qPCR-F	TAACGTGGCCAAAATGATGC	
PP2A3(At1g13320)-qPCR-R	GTTCTCCACAACCGCTTGGT	
SFP (At2g28390)-qPCR-F	AACTCTATGCAGCATTTGATCCACT	
SFP (At2g28390)-qPCR-R	TGATTGCATATCTTTATCGCCATC	

XND1-qPCR-F1	AGCCGTGCTTCTAGTTCTAGTC
XND1-qPCR-R1	GAGTTCTGTCCCATCATCGTCT
XND1-qPCR-F2	TTCCCTCCCGGATTCCTCTT
XND1-qPCR-R2	ACCCACTTGCTATAATCGGGTT
XND1-qPCR-F3	TTGCAGTGAGGAGGAAGACG
XND1-qPCR-R3	CTCTTCTTGGGTGCTTCTGTCT
PIP1;1-qPCR-F	CTGGCCTTGTCCTTAGTTGCTTC
PIP1;1-qPCR-R	TCTCCTTTGGAACTTCTTCCTTG
PIP1;2-qPCR-F	TCCTCTTCTTTGCCTAATGGAGAC
PIP1;2-qPCR-F	AGTTGCCTGCTTGAGATAAAC
PIP1;3-qPCR-F	GCTGTGGATGATCTGGTTTTATCG
PIP1;3-qPCR-R	GCCGAAACAATATGGATCTTACTC
PIP1;4-qPCR-F	CTCTGAAGTCTAAGGTGATTAGTGC
PIP1;4-qPCR-R	CAACCCGAGAACTTGATGTTGA
PIP1;5-qPCR-F	TGTTTCCTATGTCATGTGTGATG
PIP1;5-qPCR-R	GTACACAATGTATTCTTCCATTGAC
PIP2;1-qPCR-F	TGTGTTTTCCACTTGCTCTTTTG
PIP2;1-qPCR-R	CACAACGCATAAGAACCTCTTTGA
PIP2;2-qPCR-F	GGCAACTTTGCTTGTAAAACTATGC
PIP2;2-qPCR-R	AGTACACAAACATTGGCATTGG
PIP2;3-qPCR-F	GAAACATATCCTCTTTTCCACTCG
PIP2;3-qPCR-R	CTCAATACACCAAACTTACATACG
PIP2;4-qPCR-F	GGATGATTACAAATGAATTAAGTGG
PIP2;4-qPCR-R	CCACATTTACAATTACACGAATGG
PIP2;5-qPCR-F	GATATGCTCTTCCCTGAGTACATC
PIP2;5-qPCR-R	AATATCTCTCCTCACCAAAGCTAG
PIP2;6-qPCR-F	TTTCGAACTAGCGAAGAGGTGAAG
PIP2;6-qPCR-R	AGACACAGTAAATGTCACTCACC
PIP2;7-qPCR-F	TGTGTAATGAGAGAGATGGTGGA
PIP2;7-qPCR-R	AGAGAAACCAAAGGCAAACGA
PIP2;8-qPCR-F	CAACCCAACCAATTGATGATTCA
PIP2;8-qPCR-R	ACATGAAAGAAAGCAACGGAC
GFP-qPCR-F1	CGTGACCACCTTCACCTACG
GFP-qPCR-R1	GGCGGACTTGAAGAAGTCGT
GFP-qPCR-F2	GAGCTGAAGGGCATCGACTT
GFP-qPCR-R2	TTCTGCTTGTCGGCCATGAT
GFP-qPCR-F3	GGGCACAAGCTGGAGTACAA
GFP-qPCR-R3	TTCTTCTGCTTGTCGGCCAT
GFP-qPCR-F4	CACTACCTGAGCACCCAGTC
GFP-qPCR-R4	GAACTCCAGCAGGACCATGT

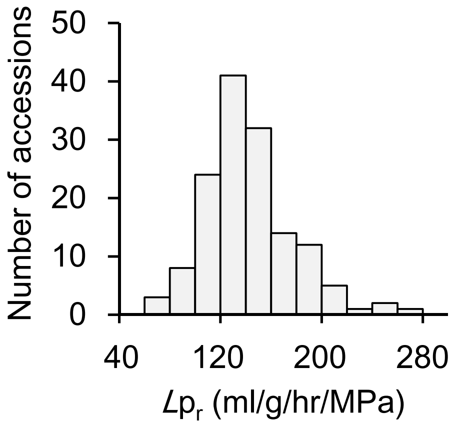

Supplementary Figure 1. Distribution of *L*p$_r$ data from 143 accessions.
Data from 4-6 individual plants per accession.

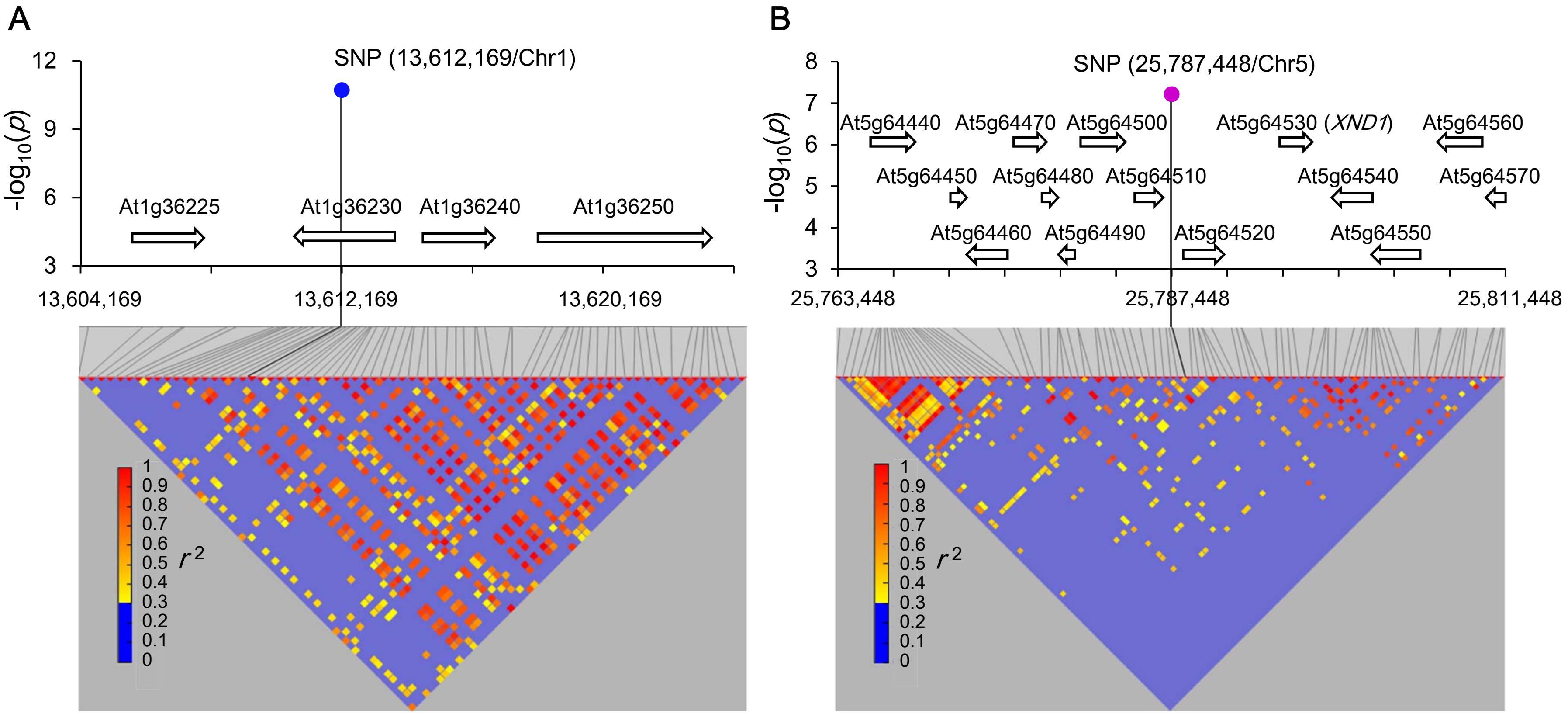

Supplementary Figure 2. Linkage disequilibrium (LD) patterns within 20 kb (A) or 48 kb (B) genomic regions surrounding the GWA peak SNPs on Chr1 (A) or Chr5 (B).

Pairwise r^2 values among polymorphisms are represented in color scale, with the GWA peak SNPs, gene structures and chromosomal positions indicated on the top.

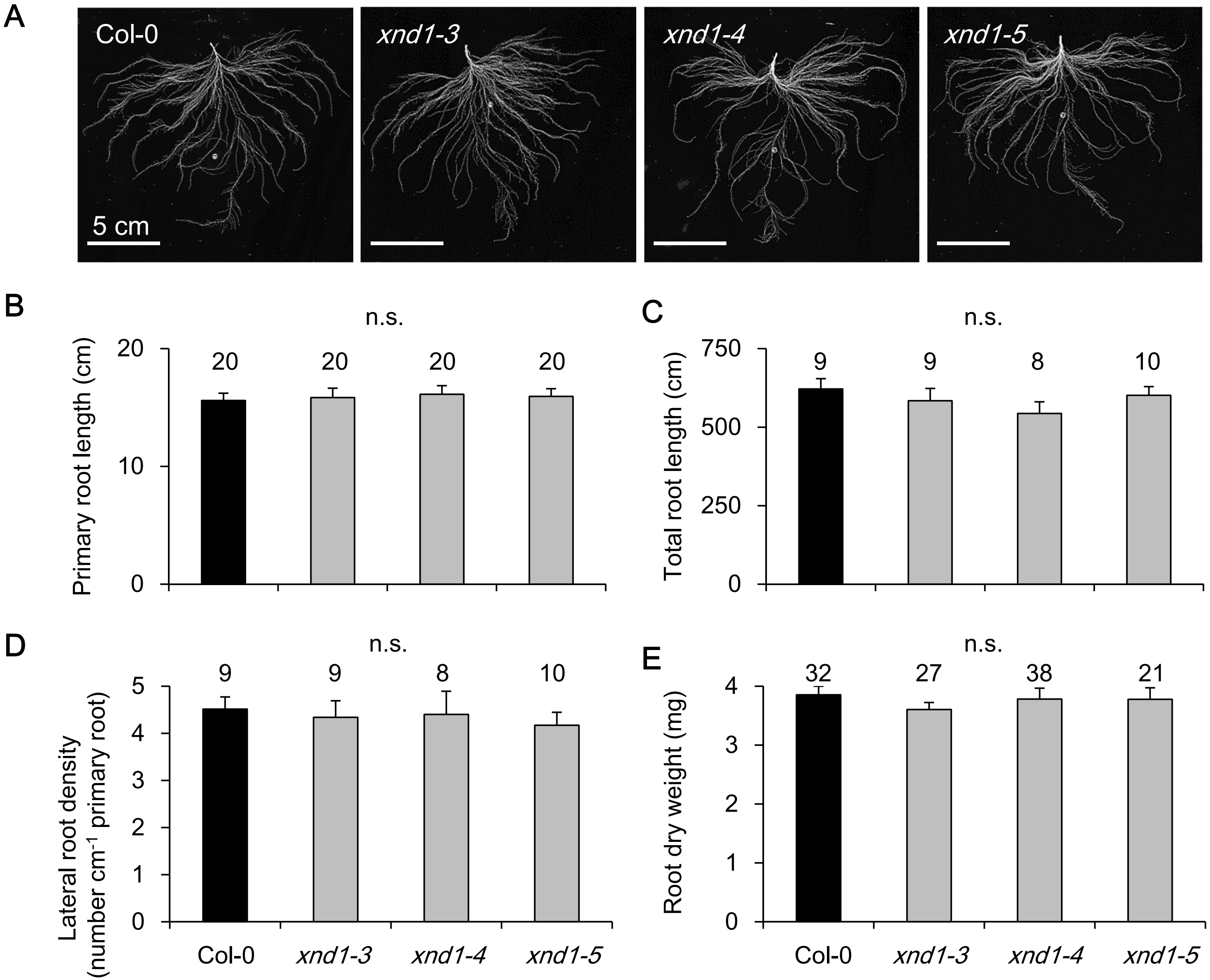

Supplementary Figure 3. Root system architecture of Col-0 and *xnd1* plants.
(**A**) Representative images of whole root systems of the indicated genotypes. Plants were grown in hydroponics for 22 days.
(**B-E**) Root morphological parameters (mean values ± SE) from the indicated genotypes and number of plants. Lateral root density was calculated from the lateral root number in the 12 cm lower segment of the primary root. One-way ANOVA ($P < 0.05$) was used to test the significance of the data (n.s. = not significant).

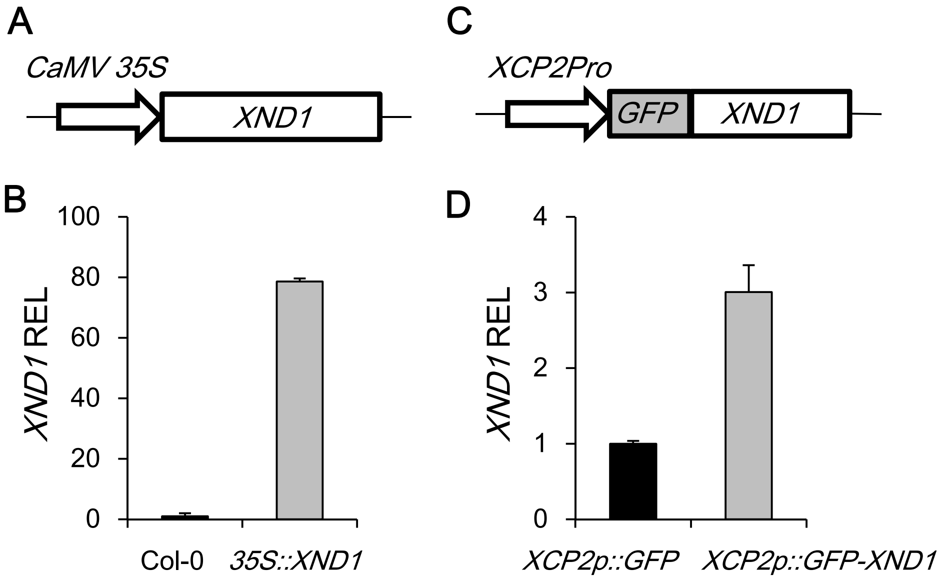

Supplementary Figure 4. Expression levels of *XND1* in ectopic expression lines.
Schematic representation of transgenic constructs (**A**, **C**; not on scale) and transcript abundance of *XND1* relative to controls in ubiquitous (*35S*::*XND1*) and xylem specific (*XCP2p*::*GFP-XND1*) expression lines (**B**,**D**). Error bars represent SE from two biological replicates.

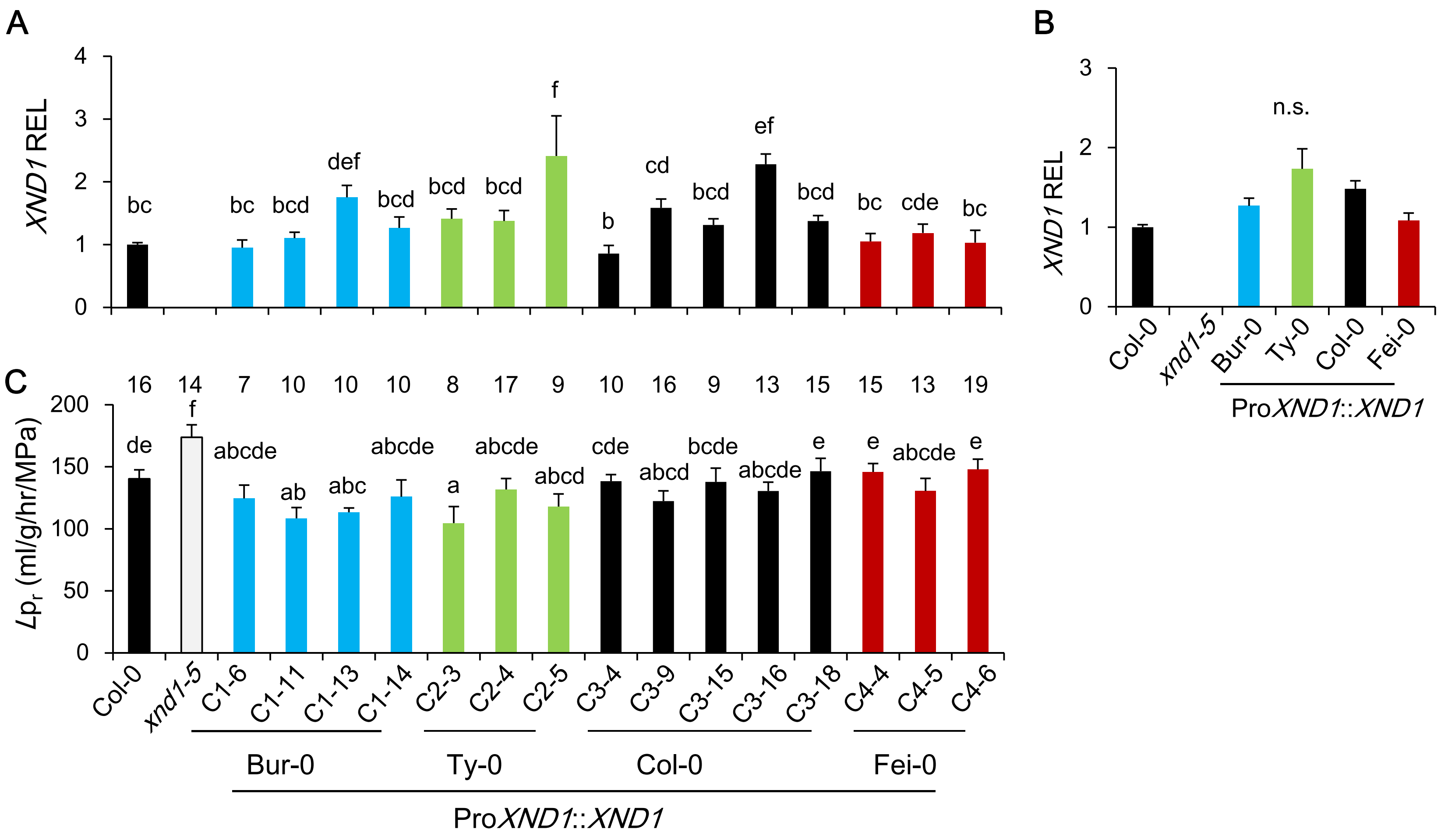

Supplementary Figure 5. Transgenic complementation of *xnd1-5* with distinct allelic forms of *XND1*.
(**A**) Transcript abundance of *XND1* in Col-0, *xnd1-5*, and individual *xnd1-5* homozygous transgenic lines expressing *XND1* genomic fragments from Bur-0, Ty-0, Col-0, or Fei-0. Mean values ± SE (n=6) from two biological replicates were normalized to transcript abundance in Col-0.
(**B**) Mean relative *XND1* transcript abundance (*XND1* REL) in Col-0, *xnd1-5* or *xnd1-5* with indicated allelic complementation. Pooled data from independent transgenic lines shown in A.
(**C**) Mean Lp_r (± SE) of corresponding lines are shown with the number of individual plants tested indicated on the top. Differences between lines in *XND1* expression (A, B) or Lp_r (C) were analyzed by one-way ANOVA (Fisher's LSD, P < 0.05; n.s.= not significant).

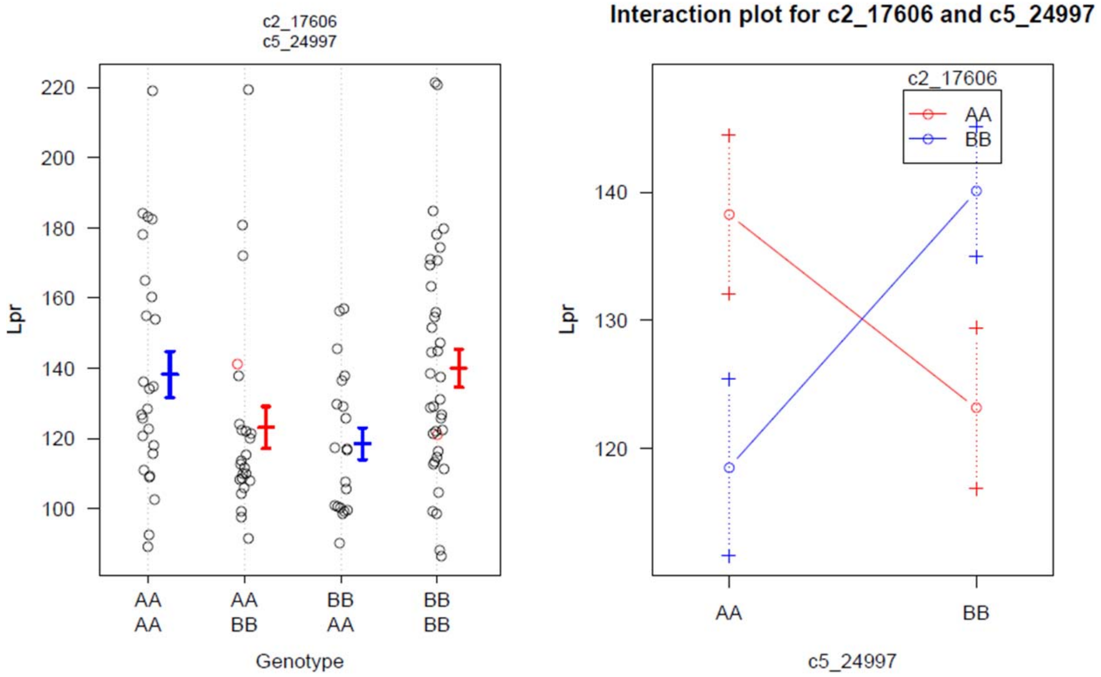

Supplementary Figure 6. Conditional epistatic interaction between two Lp_r QTL in a Bur-0 x Col-0 cross. Lp_r data presented in Shahzad et al., 2016, were used for the detection of epistatic QTLs using an Epistat program[1].The two QTLs map on Chr2 around marker c2_17606 (51.2 cM) and Chr5, around marker c5_24997 (89.6 cM). The figure shows interaction plots for Lp_r between these two genomic regions. Allelic forms: A, Col-0; B, Bur-0. Note that *XND1* is located at 25.795 Mb on Chr 5.

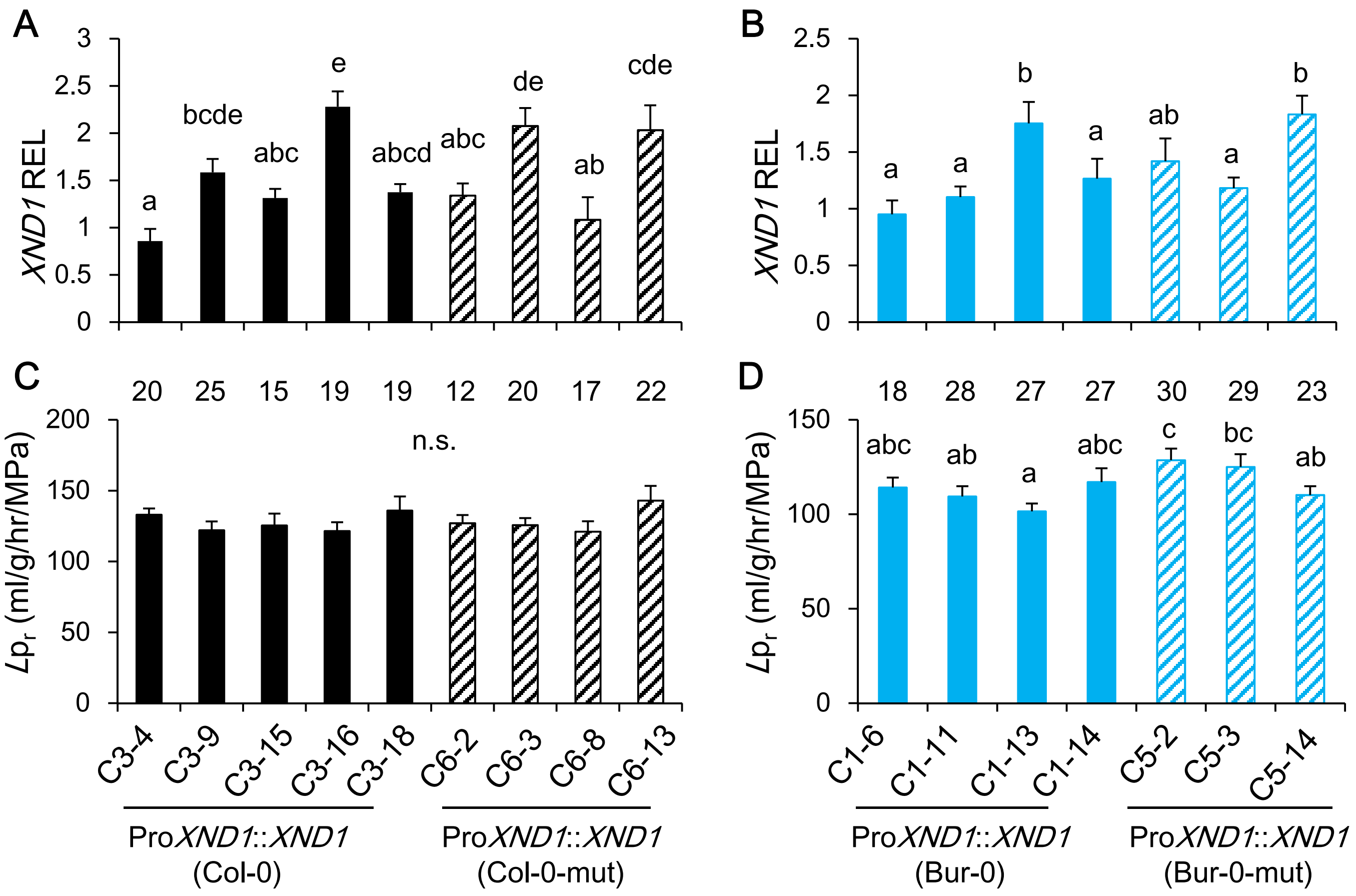

Supplementary Figure 7. Transgenic complementation of *xnd1-5* with allelic forms of *XND1*, either wild-type or with a site-directed mutation at SNP_{UTR}.

(**A**, **B**) Transcript abundance of *XND1* in *xnd1-5* homozygous transgenic lines expressing the Bur-0, Bur-0-mut, Col or Col-0-mut allelic forms of *XND1*. Mean values ± SE (n=6) based on two biological replicates were normalized to transcript abundance in Col-0.

(**C, D**) Lp_r of same transgenic lines as in A and B. Mean values ± SE from the indicated number of plants are shown. Differences between lines were analyzed in each panel using a one-way ANOVA (Fisher's LSD, $P < 0.05$; n.s.= not significant).

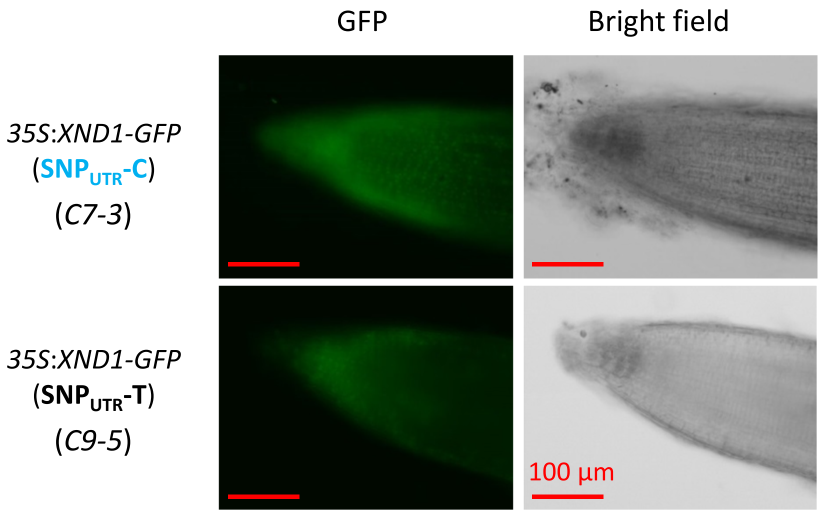

Supplementary Figure 8. Transgenic expression of *XND1-GFP*.
A *XND1-GFP* construct placed under the control of a *CaMV 35S* promoter, with either C or T at SNP_{UTR}, was introduced into *xnd1-5* and roots of hydroponically grown plants were checked for GFP fluorescence intensity. Data from two representative lines (*C7-3* and *C9-5;* see Supplementary Figure 9).

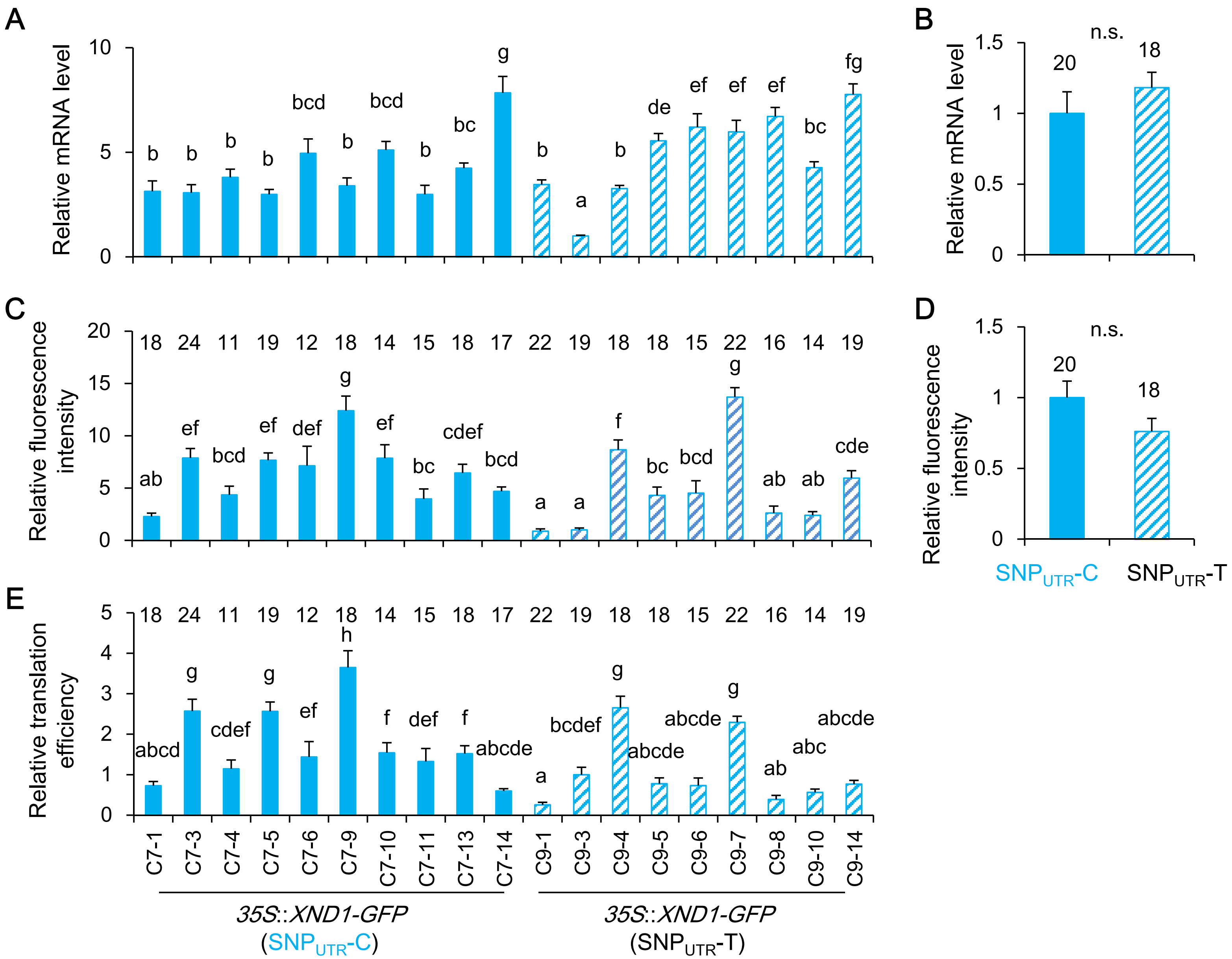

Supplementary Figure 9. SNP_{UTR} of *XND1* contributes to translational efficiency.
(**A**) Transgenic *xnd1-5* lines expressing a *XND1-GFP* fusion under the control of a *CaMV 35S* promoter, with either C or T at SNP_{UTR}, were characterized for transcript abundance of *GFP* in roots. Mean values ± SE (n=12) for each individual lines based on two biological replicates were normalized to transcript abundance in line *C9-3*.

Supplementary Figure 9 (continued)
(**B**) Pooled data from transgenic lines shown in A. Mean values ± SE based on total number of lines and repeats are indicated on the top and were normalized to the data for SNP_{UTR} -C.
(**C**) Relative fluorescence intensity of the same transgenic lines, as measured in root tips using a fluorescence microscope. Mean values ± SE based on total number of plants indicated on the top were normalized to the mean fluorescence signal in line *C9-3*.
(**D**) Pooled data from transgenic lines shown in B. Same conventions as in (B).
(**E**) Relative translation efficiency (GFP intensity / mRNA level) was calculated based on data from A and C. Differences between lines were analyzed in A, C and E using a one-way ANOVA (Fisher's LSD, $P < 0.05$). Student's *t* test was used to assess the statistical significance of pooled data (B, D).

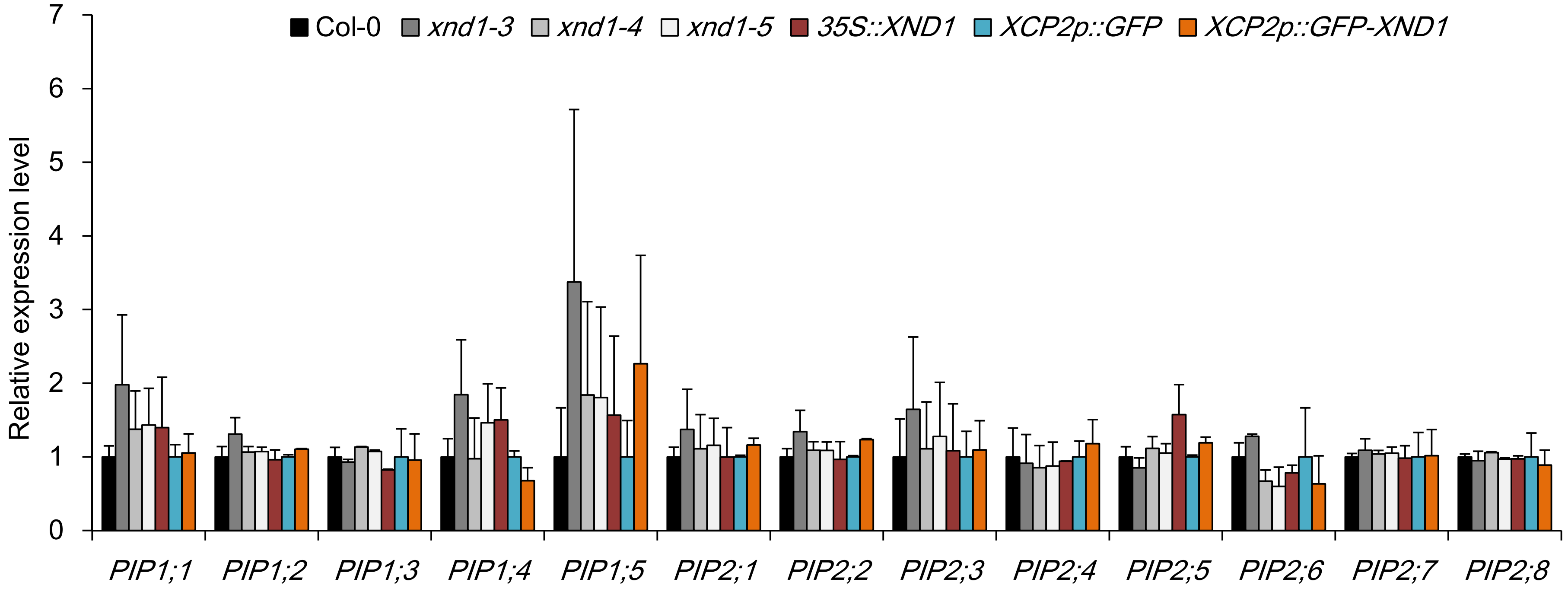

Supplementary Figure 10. Transcript abundance of *PIP* genes in *xnd1* T-DNA insertion mutants and *XND1* ectopic expression lines.

Transcript abundance of indicated *PIP* genes was determined by qRT-PCR in roots of control lines (Col-0, *XCP2p::GFP*), *xnd1* T-DNA insertion lines (*xnd1-3*, *xnd1-4*, *xnd1-5)* and two *XND1* ectopic expression lines (*35S::XND1*, *XCP2p::GFP-XND1*). Data (means ± SE) cumulated from two biological replicates were expressed relative to *XND1* transcript abundance in control lines.

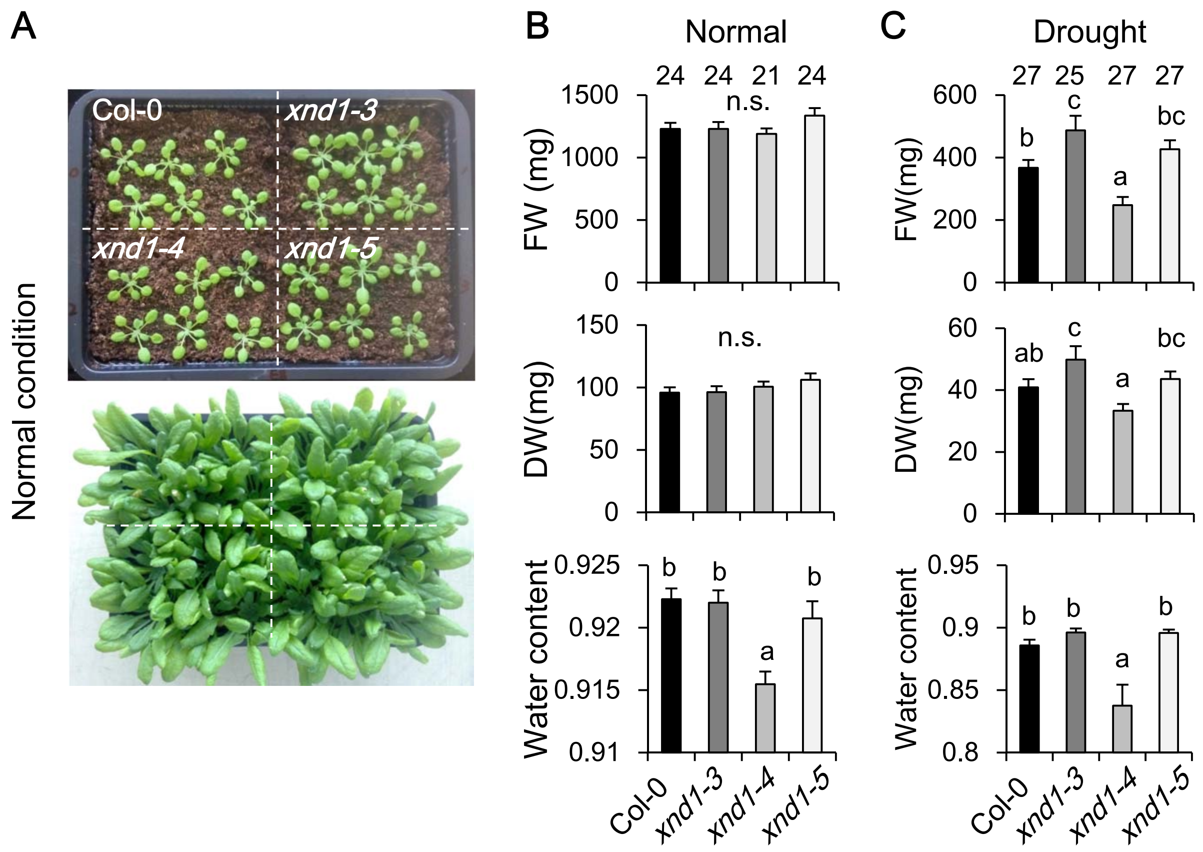

Supplementary Figure 11. Growth of Col-0 and *xnd1* plants under water sufficient or limiting conditions.
(**A**) Shoot phenotypes of 22-day-old (upper panel) and 51-day-old (lower panel) Col-0 and *xnd1* plants grown under non-limiting irrigation.
(**B**, **C**) Fresh weight (FW), dry weight (DW) and water content of shoots of Col-0 and *xnd1* plants under non-limiting irrigation (B) or after 24 days of water deficit and 5 days of rewatering (C). The figure C shows the raw data (means ± SE from the indicated number of plants and 4 biological replicates) used, prior to normalization, to construct Figure 6. Differences between lines were analyzed in each panel using a one-way ANOVA (Fisher's LSD, $P < 0.05$; n.s.= not significant).

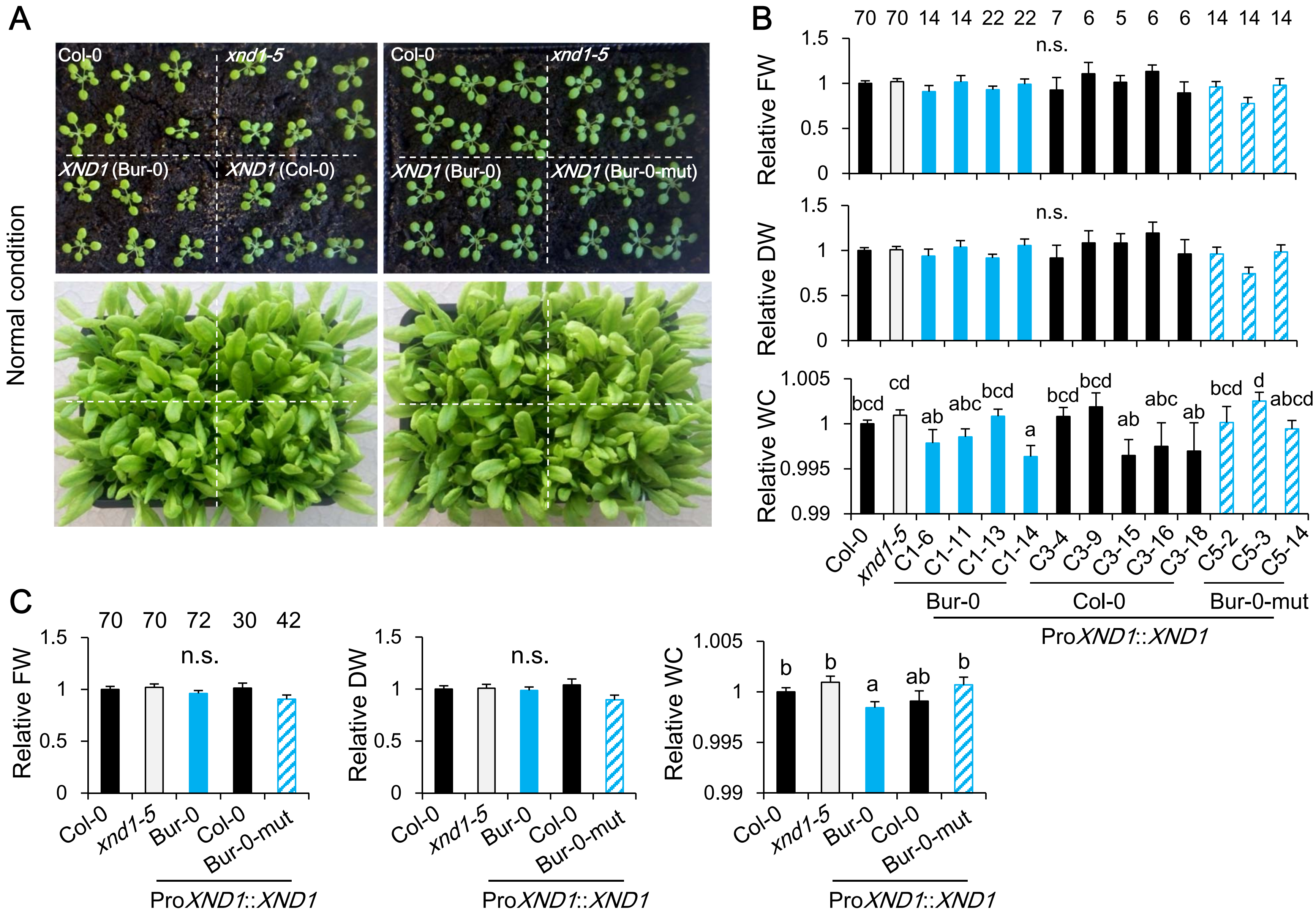

Supplementary Figure 12. Growth under water sufficient conditions of Col-0, *xnd1-5*, and *xnd1-5* expressing distinct allelic forms of *XND1*.
(A) Rosette phenotype of 22- (upper panels) and 51- (lower panels) day-old plants of indicated genotypes.

Supplementary Figure 12 (continued)

(B) Relative FW, DW and water content (WC) in shoots of same genotypes. Three to five independent transgenic lines were measured for each allelic form.

(C) Mean values ± SE for each allelic form were calculated from data of independent transgenic lines described in B and are shown with sample size indicated on the top. One-way ANOVA (Fisher's LSD, $P < 0.05$) was used to test the significance of the data (n.s.= not significant).

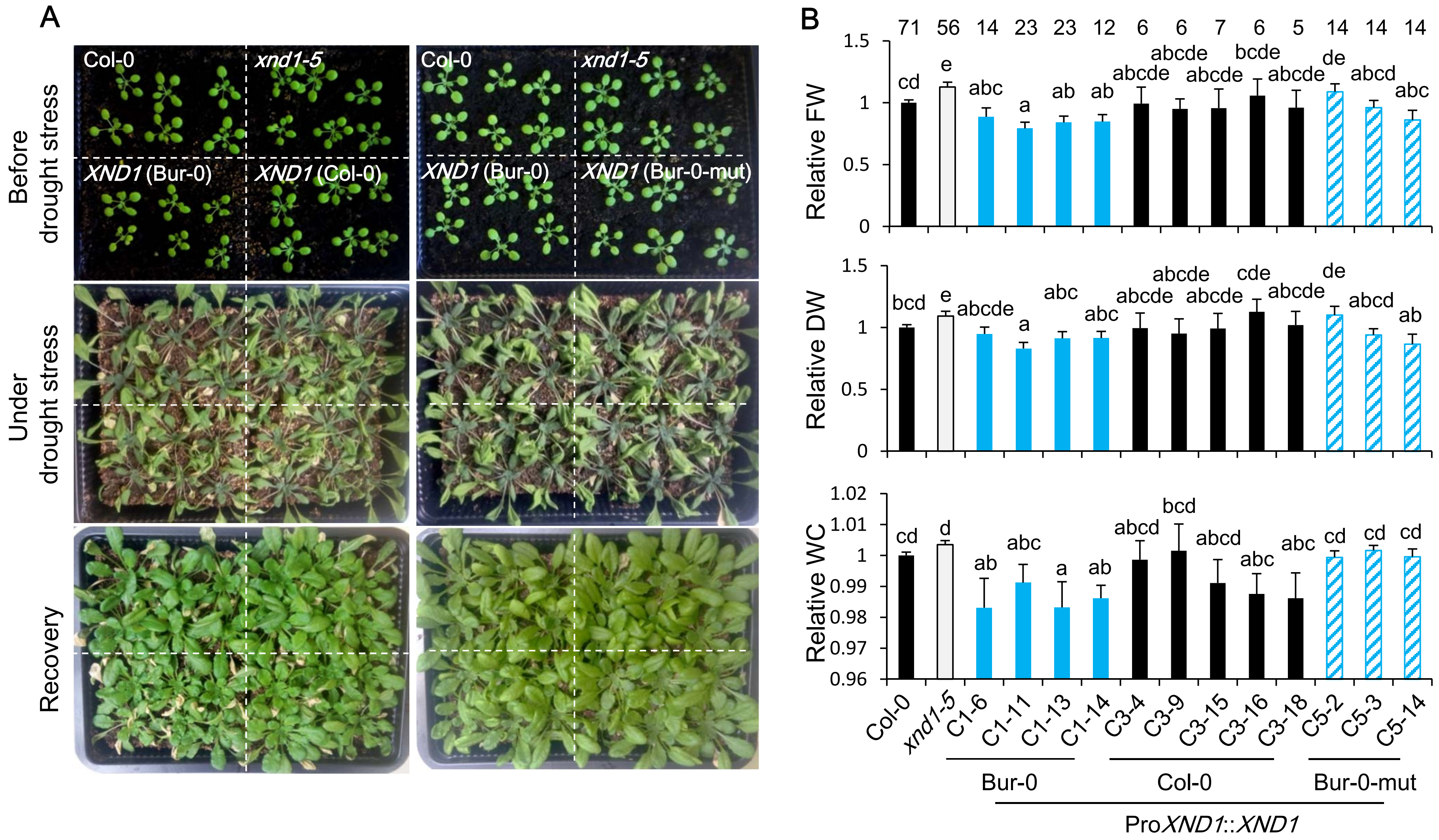

Supplementary Figure 13. Growth under water limiting conditions of Col-0, *xnd1-5*, and *xnd1-5* plants expressing distinct allelic forms of *XND1*.

(A) Rosette phenotypes of plants of indicated genotypes at 22 days post-germination (upper panel), after water deprivation for 24 additional days (middle panel), and at 5 days after rewatering (lower panel).

(B) Relative FW, DW and WC in shoots of indicated genotypes after rewatering. Mean values ± SE are shown with the number of plants indicated on the top. One-way ANOVA (Fisher's LSD, $P < 0.05$) was used to test the significance of the data.

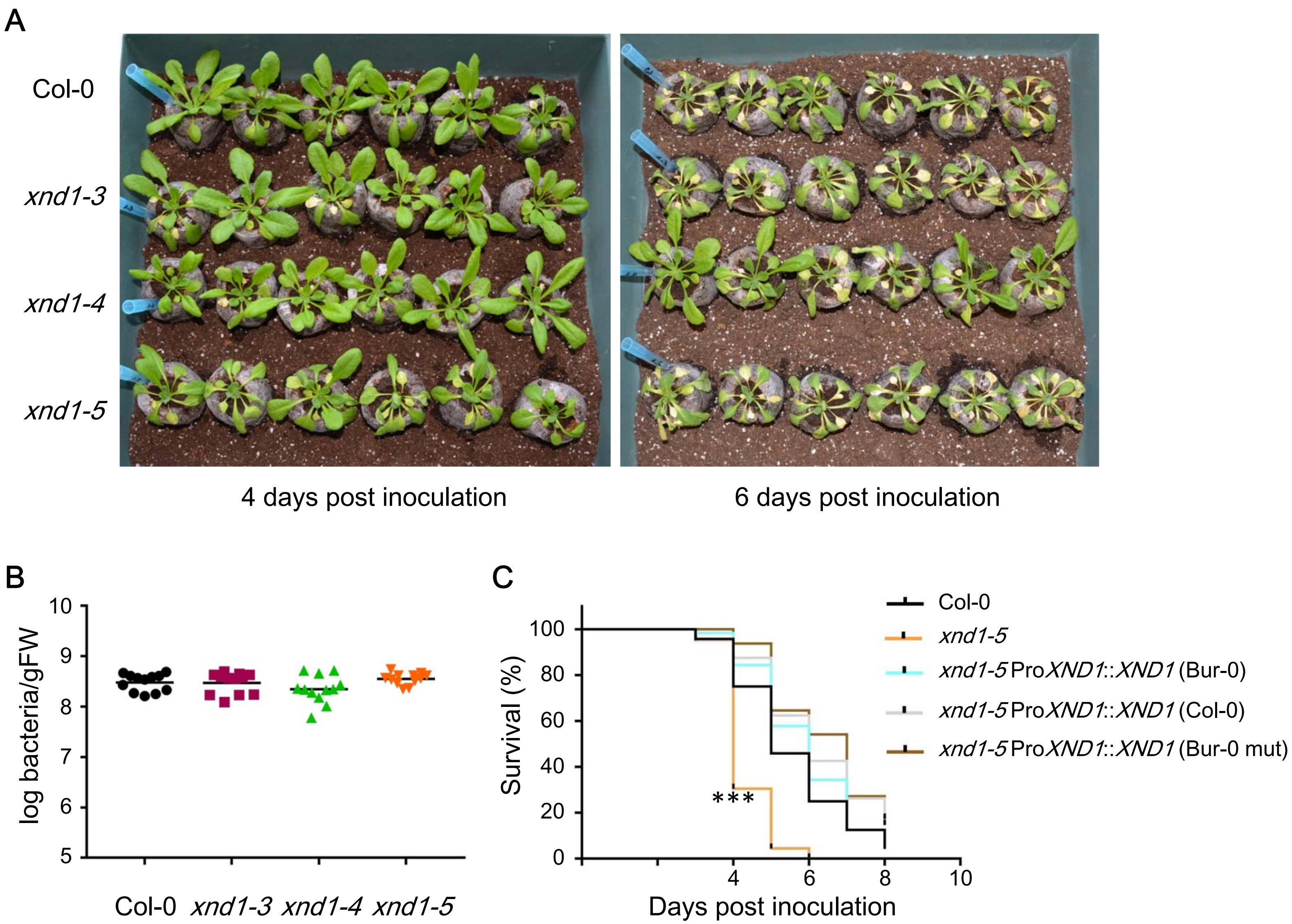

Supplementary Figure 14. *R. solanacearum* infection assays in wild type (Col-0) and *xnd1* mutant plants, and in *xnd1-5* plants expressing distinct allelic forms of *XND1*.
(**A**) Representative pictures Col-0 and *xnd1 plants* at 4 and 6 days after root inoculation by *R. solanacearum*.

Supplementary Figure 14 (continued)
(**B**) *in planta* growth measurement of *R. solanacearum* at 4 days after inoculation of the indicated genotypes. Each dot represents a replicate of two plants and the black line indicates the mean of all the replicates. Cumulated data from 4 independent biological repeats. No significant difference between genotypes was observed (Mann-Whitney test; $P < 0.05$).
(**C**) Survival response of the indicated genotypes to *R. solanacearum* inoculation. Plants were inoculated through root dipping and plant survival (in %) was scored at the indicated days post-inoculation using a Kaplan–Meier survival analysis. Gehan-Breslow-Wilcoxon test indicates that the *xnd1-5* curve is significantly different from the Col-0 curve ($P = 0.0006$) whereas the curves obtained after complementation of *xnd1-5* with the Col-0, Bur-0, and Bur-0-mut alleles are not ($P = 0.1712$, $P = 0.0533$, and $P = 0.1726$, respectively). The survival curves were deduced from 24 plants for Col-0 and *xnd1-5*, 64 plants for *xnd1-5* Pro*XND1*::*XND1* (Bur-0), 80 plants for *xnd1-5* Pro*XND1*::*XND1* (Col-0) and 48 plants for *xnd1-5* Pro*XND1*::*XND1* (Bur-0-mut). Two fully independent biological replicates were performed with similar results.

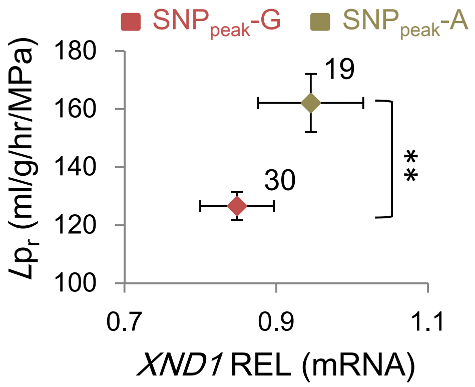

Supplementary Figure 15. Lack of correlation between *XND1* transcript abundance and *L*p$_r$ in natural accessions.

Accessions were grouped according to their genotype (G or A) at the GWA peak SNP on Chr 5 (position 25,787,448).

SUPPLEMENTARY REFERENCE

1. Chase, K., Adler, F. R. & Lark, K. G. Epistat : a computer program for identifying and testing interactions between pairs of quantitative trait loci. Theor. Appl. Genet. 94, 724-730 (1997).
