# Supplementary material for: Natural variation at XND1 impacts root hydraulics and trade-off for stress responses in Arabidopsis
Source: Nat Commun. 2018 Sep 24;9:3884. doi: 10.1038/s41467-018-06430-8 (PMC6155316; doi:10.1038/s41467-018-06430-8)
Supplement: Supplementary file 2 — Description of Additional Supplementary Files [file 41467_2018_6430_MOESM2_ESM.pdf]

DESCRIPTION OF ADDITIONAL SUPPLEMENTARY FILES:

Supplementary Data 1. *L*p$_r$ characterization of natural accessions.
The data support the GWA analysis presented in Figure 1A to 1C.

Supplementary Data 2. Accessions used for *XND1*-based local association and haplogroup analysis.
The data support the GWA analysis presented in Figure 2A to 2C.

Supplementary Data 3. *L*p$_r$ data for transgenic complementation lines of *xnd1-5* with distinct allelic forms of *XND1*.
These data correspond to the plots of Figure 2D and Supplementary Figure 5C.

Supplementary Data 4. *L*p$_r$ data for transgenic complementation lines of *xnd1-5* with allelic forms of *XND1*, either wild-type or with a site-directed mutation at SNP_{UTR}.
These data correspond to the plots of Figure 3C and 3F and Supplementary Figure 7C and 7D.
